# Genes and functions from breast cancer signatures

**DOI:** 10.1186/s12885-018-4388-4

**Published:** 2018-04-27

**Authors:** Shujun Huang, Leigh Murphy, Wayne Xu

**Affiliations:** 1 Research Institute of Oncology and Hematology, CancerCare Manitoba, 675 McDermot Ave, Winnipeg, Manitoba R3E 0V9 Canada; 2 Department of Biochemistry and Medical Genetics, Faculty of Health Sciences, University of Manitoba, Winnipeg, Manitoba R3E 0J9 Canada; 3 College of Pharmacy, Faculty of Health Sciences, University of Manitoba, Winnipeg, Manitoba R3E 0J9 Canada

**Keywords:** Breast cancer, Multi-gene signature, Common gene, Common function

## Abstract

**Background:**

Breast cancer is a heterogeneous disease and personalized medicine is the hope for the improvement of the clinical outcome. Multi-gene signatures for breast cancer stratification have been extensively studied in the past decades and more than 30 different signatures have been reported. A major concern is the minimal overlap of genes among the reported signatures. We investigated the breast cancer signature genes to address our hypothesis that the genes of different signature may share common functions, as well as to use these previously reported signature genes to build better prognostic models.

**Methods:**

A total of 33 signatures and the corresponding gene lists were investigated. We first examined the gene frequency and the gene overlap in these signatures. Then the gene functions of each signature gene list were analysed and compared by the KEGG pathways and gene ontology (GO) terms. A classifier built using the common genes was tested using the METABRIC (Molecular Taxonomy of Breast Cancer International Consortium) data. The common genes were also tested for building the Yin Yang gene mean expression ratio (YMR) signature using public datasets (GSE1456 and GSE2034).

**Results:**

Among a total of 2239 genes collected from the 33 breast cancer signatures, only 238 genes overlapped in at least two signatures; while from a total of 1979 function terms enriched in the 33 signature gene lists, 429 terms were common in at least two signatures. Most of the common function terms were involved in cell cycle processes. While there is almost no common overlapping genes between signatures developed for ER-positive (e.g. 21-gene signature) and those developed for ER-negative (e.g. basal signatures) tumours, they have common function terms such as cell death, regulation of cell proliferation. We used the 62 genes that were common in at least three signatures as a classifier and subtyped 1141 METABRIC cases including 144 normal samples into nine subgroups. These subgroups showed different clinical outcome. Among the 238 common genes, we selected those genes that are more highly expressed in normal breast tissue than in tumours as Yang genes and those more highly expressed in tumours than in normal as Yin genes and built a YMR model signature. This YMR showed significance in risk stratification in two datasets (GSE1456 and GSE2034).

**Conclusions:**

The lack of significant numbers of overlapping genes among most breast cancer signatures can be partially explained by our discovery that these signature genes represent groups with similar functions. The genes collected from these previously reported signatures are valuable resources for new model development. The subtype classifier and YMR signature built from the common genes showed promising results.

**Electronic supplementary material:**

The online version of this article (10.1186/s12885-018-4388-4) contains supplementary material, which is available to authorized users.

## Background

Breast cancer is a heterogeneous disease. Patients at the same stage or in the same molecular subtype can exhibit different clinical prognosis or different benefit from systemic therapy. Personalized medicine is urgently needed for best breast cancer care. For this reason multi-gene signatures have been extensively studied to provide prognostic and predictive information for breast cancer treatment. Today more than 30 different signatures have been reported [1–42]. Several signatures have become commercially available including the 70-gene signature (MammaPrint) [1], the 21-gene signature (OncotypeDx) [4], the 97-gene genomic grade index (GGI) [15], the EndoPredict assay [33], the breast cancer index [21], and the PAM50 assay [3]. The 70-gene signature [1] and the 21-gene signature [4] can distinguish patients with different risk for relapse and patients with high risk benefit more from adjuvant chemotherapy (CT) than patients with low risk. The 97-gene genomic grade index [15] divides classic histologic grade into low and high risk patients. The breast cancer index [21] divides patients into groups with different risk of recurrences, and low-risk patients have high responsiveness to adjuvant tamoxifen therapy. The EndoPredict [33] predicts the high-risk or low-risk groups of relapse, indicating CT/no CT. The PAM50 assay [3] is a classifier for subtyping breast cancer into five subtypes: luminal A (LumA), luminal B (LumB), HER2-enriched (Her2), basal-like (Basal) and normal-like (Normal). PAM50 assay [3] also assesses a patient’s risk of distant recurrence of disease and likelihood of efficacy from neoadjuvant CT. These commercialized signatures have proved to work well in hormone receptor (HR)-positive breast cancers. Several signatures have also been reported to define patients with a good prognosis within the ER-negative tumour cohorts [18, 26]. A number of signatures were derived to predict clinical outcome for triple-negative breast cancers (TNBC) or basal breast cancers [31, 35, 36, 38, 41]. Signatures derived from HER2-positive cohorts are used usually for predicting trastuzumab response [28, 39]. Several signatures have been developed for predicting docetaxel response [2, 6, 16].

However, signatures derived for similar tumour cohorts for similar purposes share little overlapping genes [20, 40]. For example, the 70-gene signature shares only three common genes with the 64-gene signature [20] while both signatures were mainly derived from ER-positive patients. A previous study using functional enrichment analysis of a limited six gene signatures showed that there was little overlap of functional categories among these six signatures [24]. However, another study showed a prognostic concordance among several gene expression signatures, suggesting potential equivalence between the signatures [43]. We hypothesized that although these signatures do not have many overlapping genes, they could share common functions or pathways.

The genes of previously reported signatures could be valuable resources for new approaches because these have been tested to be more or less associated with clinical outcomes in the original studies. Some studies have pooled previously reported datasets to develop a new signature [24, 44] and one study reported that combining several signature gene sets improved survival prediction from breast cancer [37]. In addition, different breast cancer datasets can be considered as resamplings from the underlying breast cancer population and the genes most frequently identified (common genes) in the separate resamplings were put forward as a ‘gold standard’ [24]. Therefore, these common genes identified from these signature genes could be valuable resources for new signature development.

Recently, we have developed a 16 Yin Yang gene mean expression ratio (YMR-16) signature for ER-positive/node-negative breast cancer based on the hypothesis that two opposing effects (Yin and Yang) could determine cancer initiation and progression [42]. These 16 genes were identified among all human genes on the Illumina gene expression microarray platform. In this study, we attempted to understand why there are so few overlapping genes among the previously reported multi-gene signatures, as well as to address our hypothesis that signatures share common functions or pathways [42, 45, 46]. We also evaluated the cohort of genes found to be common from multiple signatures in our Yin Yang gene Mean expression Ratio (YMR) model [42] and as a subtyping classifier.

## Methods

### Signature genes and test data

**Fig. 1:**
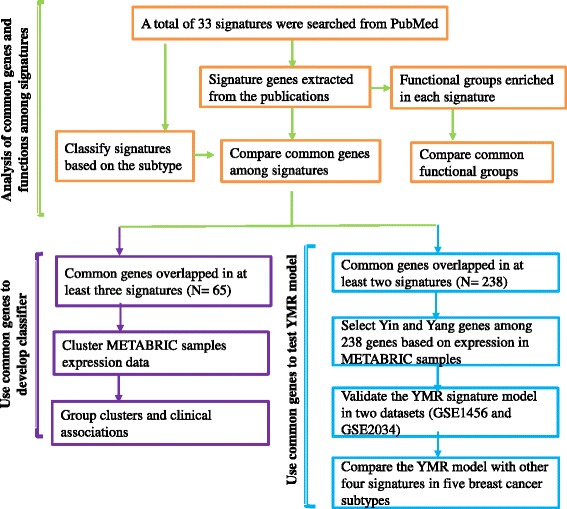
Project schema Three components: common genes and common functions analyses, common gene classifier test, and YMR model test using common genes

Our approach is depicted in Fig. 1. We searched PubMed for breast cancer gene signatures or classifiers (Table 1, Additional file 1: Table S1), and collected the gene lists from the original publications. We used HUGO gene symbols to build the gene lists. If only probe IDs were available the gene symbols for the probe IDs were retrieved from the corresponding annotations for the platforms. Since some studies used different gene alias names, we uniformly identified all gene names as official gene symbols by a custom R script. Signatures without gene symbols or probe IDs available were excluded.

**Table 1:** Published signatures included in the analysis

Full name	Short name^a^	gene #	Extract #	Cov %^b^	Used for^c^	Platform^d^	Purpose^e^		
							Prognosis	Predict	Subtyping
70-gene signature [1]	Mamma	70	66	100	ER+	Agilent Hu25K	yes	yes	no
21-gene signature [4]	RS	16	16	100	ER+	RT-PCR	yes	yes	no
97-gene genomic grade index [15]	GGI97	97	94	100	ER+	Affymetrix U133A	yes	yes	no
8-gene genomic grade index [27]	GGI8	4	4	100	ER+	qRT-PCR	yes	yes	no
EndoPredict assay [33]	Endo	8	8	100	ER+	qRT-PCR	yes	yes	no
Breast cancer index [21]	BCI	7	7	100	ER+	qRT-PCR	yes	yes	no
HOXB13:IL17 BR ratio [14]	HI	2	2	100	ER+	Agilent Arcturus 22 k	yes	yes	no
IHC4 Score [32]	IHC4	4	4	100	ER+	IHC	yes	yes	no
14-gene metastasis score [23]	MS14	14	14	100	ER+	RT-PCR	yes	no	no
8-gene score [29]	SMS	8	8	100	ER+	qRT-PCR	yes	no	no
PAM50 assay [3]	PAM50	50	50	100	BC	Agilent human 1Av2	yes	yes	yes
76-gene signature [10]	Wang	76	71	100	BC	Affymetrix U133A	yes	yes	no
64-gene expression signature [9]	Pawitan	64	48	75	BC	Affymetrix U133	yes	yes	no
32-gene p53 status signature [7]	p53	32	21	100	BC	Affymetrix U133 A or B	yes	yes	yes
Cell cycle pathway signature [20]	CCPS	26	26	100	BC	Affymetrix U95 Av2 or U133	yes	no	no
127-gene classifier [24]	Robust	127	127	100	BC	Affymetrix microarray	yes	no	no
26-gene stroma-derived prognostic predictor [19]	SDPP	26	26	100	BC	Agilent 44 k	yes	no	no
54-gene lung metastasis signature [8]	LM	54	54	100	BC	Affymetrix U133A	yes	no	no
186-invasivenessgene signature [17]	IGS	186	186	100	BC	Affymetrix U133 A or B	yes	no	no
92-gene predictor [2]	Chang	92	86	100	BC	Affymetrix U95 Av2	no	yes	no
85-gene signature [6]	Iwao	85	73	100	BC	ATAC-PCR	no	yes	no
512-gene signature [16]	Olaf	512	355	69	BC	Human Oligo set 2.1	no	yes	no
7-gene immune response module [18]	IR7	7	7	100	ER-	Agilent and Affymetrix microarray	yes	no	yes
T-cell metagene [26]	Tcell	50	50	100	ER-	Affymetrix U133A	yes	no	no
Multigene HRneg/Tneg signature [31]	Multigene	14	14	100	TNBC	Affymetrix U133A	yes	no	no
26-gene signature [35]	Novel1	26	20	100	TNBC	Affymetrix U133	yes	yes	no
264-gene signature [35]	Novel2	264	225	100	TNBC	Affymetrix U133	yes	no	no
B-cell:IL8 ratio [41]	Bcell	22	20	100	100 TNBC	Affymetrix U133A or U133 Plus 2.0	yes	no	no
MAGE-A [38]	MAGEA	2	2	100	TNBC	Affymetrix U133	yes	no	no
368-gene medullary breast cancer like signature [36]	MBC	368	368	100	TNBC	Affymetrix or Agilent aaray	yes	no	yes
158-gene HER2-derived prognostic predictor [30]	HDPP	158	158	100	HER2+	SWEGENE H_v2.1.1 55 K	yes	no	yes
GCNs of MET and HGF [39]	GCN	2	2	100	HER2+	Fluorescence in situ hybridisation (FISH)	no	yes	No
28-gene expression profile [28]	Vegran	28	27	100	HER2+	Affymetrix microarray	no	yes	no

^a^The short name for the full name used in this paper

^b^For some signatures with 100% coverage (all signature genes were found in data set), the extracted gene No (Extract# column) is less than the reported gene No (gene# column) because some genes are duplicated with different probe names within a signature

^c^The subtype the signatures are developed for: ER+, the signature is used for ER-positive breast cancer; ER-, the signature is used for ER-negative breast cancer; uc-BC, the signature is used for un-classified breast cancer with mixed subtypes; TNBC, the signature is used for TNBC or basal breast cancer; HER2+, the signature is used for HER2-positive breast cancer

^d^The experimental platform used for developing the signatures

^e^The clinical purpose of these signatures: prognosis, the signature can be used for prognosis; prediction, the signature can be used for predicting the response to treatment or drug; subtyping, the signature can be used for further subtyping breast cancers

To test the usefulness of these previously reported signature genes in new prognostic signature development, we used three cohort data sets. One was The Molecular Taxonomy of Breast Cancer International Consortium (METABRIC) data [47]. A total of 1141 samples, including 997 tumours and 144 controls, were included. The other two were from NCBI’s Gene Expression Omnibus (GEO) with the accession number of GSE1456 and GSE2034. GSE1456 includes 159 tumours with gene expression data and clinical information. GSE2034 comprises data from 107 tumour samples. All these datasets include clinical follow-up of disease-specific survival (DSS) or recurrence free survival (RFS) time. In addition, 5 datasets from Bioconductor libraries: breastCancerMAINZ (GSE11121), breastCancerTRANSBIG (GSE7390), breastCancerUPP (GSE3494), breastCancerUNT (GSE2990), breastCancerNKI were used for signature comparisons.

### Comparing all signature genes

We first compiled a matrix with each of all signature genes against each of the 33 signatures (Additional file 2: Table S2). Then a signature number for each gene was counted (Additional file 3: Table S3). We also generated a matrix of 33 signatures by 33 signatures with the common genes for each signature (Additional file 4: Table S4). This matrix allowed us to view the heat map of common gene frequencies among all signatures using Partek Genomics Suite software version 6.6 (Partek Inc., St. Louis, MO, USA).

### Comparing function groups enriched by signatures

To determine what biological functions or pathways are enriched in a signature gene list, we utilized the Database for Annotation, Visualization and Integrated Discovery (DAVID) for gene function enrichment analysis. We selected GO_term Biological processes and KEGG pathways to define function enrichment with the default settings (EASE *p*-value less than 0.1). Similar approaches to gene comparison were applied to the function distribution analysis (Additional file 5: Table S5 and Additional file 6: Table S6), and the common function analysis (Additional file 7: Table S7). We also viewed the common functions among all signatures by generating a frequency heat map.

### Clustering breast cancers by common signature genes

We tested if the common genes from multiple signatures could be used to develop breast cancer subtype classifiers. A total of 65 genes were common in three or more signatures. Sixty-two of these genes were matched to METABRIC data set and used to cluster the 1141 METABRIC samples including 144 normal samples by 2D Euclidean clustering with complete linkage settings. We then examined the clinical outcome of each cluster of patients. Kaplan-Meier analysis on the METABRIC patient groups was performed.

### Developing YMR signature from common signature genes

We also evaluated if the previously identified signature genes could be used for new prognostic signature development. In this study, we developed and tested the YMR signature. We first selected the Yin genes that were more highly expressed in tumours than in normal breast tissue samples and the Yang genes that were more highly expressed in normal than in tumour tissue samples. Two hundred thirty-eight common genes were found in two or more signatures and 220 of them could be matched to the METABRIC database. We used the 1141 METABRIC samples data to identify the Yin and Yang genes from the 220 common gene groups by 2D clustering. These Yin and Yang genes were applied to the YMR model. We then validated the YMR model using GSE1456 and GSE2034, respectively. Patients in the two datasets were divided into four groups with low, inter-low, inter-high, high risk of death according to the YMR scores. Kaplan-Meier analysis was then performed on the four patient groups using the available clinical information. The associated *p*-values generated from log-rank test in Kaplan-Meier analysis were used to represent the statistical significance of differential survival probabilities between different patient groups. We used Bioconductor geneFu package [48] to compare the YMR signature developed from the common signature genes with other prognostic signatures including our previous YMR-16, MammaPrint, OncotypeDx and Multigene HRneg/Tneg signature. Risk stratifications were evaluated within each of the five different untreated subtypes including ER-positive/node-negative, luminal A, luminal B, HER2-enriched and basal-like of 5 datasets from Bioconductor libraries: breastCancerMAINZ (GSE11121), breastCancerTRANSBIG (GSE7390), breastCancerUPP (GSE3494), breastCancerUNT (GSE2990), breastCancerNKI.

## Results

### 33 signatures

A total of 33 published breast cancer gene signatures were included in the study (Table 1, Additional file 1: Table S1). More than half of these signatures (18/33) were developed using the Affymetrix microarray platform. We classified signatures into three types based on their applications in the original studies: clinical prognosis (prognosis), prediction of treatment benefit (prediction), and subtyping breast cancers (subtyping), though many signatures can be used for more than one application. We also classified signatures into four categories based on the population they were used for: ER-positive breast cancer (ER+), TNBC or basal breast cancer (TNBC), HER2-positive breast cancer (HER2+), un-classified breast cancer patients (uc-BC) or mixed types. IR7 was used for ER-negative breast cancer while Tcell was for ER-negative or HER2-positive breast cancer, so we classified Tcell to both the TNBC and the HER2+ signature group and IR7 to the TNBC group. Therefore, the number of signatures included in ER+, uc-BC, TNBC and HER2+ groups are 10, 12, 8 and 4, respectively. The 10 signatures Mamma, RS, GGI97, GGI8, Endo, HI, BCI, IHC4, MS14 and SMS were mostly applied to ER-positive breast cancers. Five of them, Mamma, RS, GGI97, Endo, and BCI, have been commercialized. GGI8 is a 4-gene version of GGI97, while HI signature was included in BCI. These signatures can be used to predict the risk for recurrence for ER-positive patients and thus inform recommendations for taking adjuvant therapy in these patients. The other 11 signatures, PAM50, Wang, Pawitan, p53, CCPS, Robust, SDPP, LM, Chang, Iwao and Olaf were used for primary or mixed breast cancer subtypes. Among these 11 signatures, PAM50 was used for subtyping, three (Chang, Iwao and Olaf) were for predicting docetaxel response while the others were mainly used for prognosis prediction. IGS was derived by comparing the gene-expression profile of CD44 + CD24−/low tumorigenic breast cancer cells with that of normal breast epithelium for predicting invasiveness.

Tcell and IR7 signatures were used to define patients with a good prognosis from ER-negative tumours. The six signatures, Multigene, Novel1, Novel2, Bcell, MAGEA and MBC, were developed to predict clinical outcome for TNBC patients or basal breast cancers. Three signatures, HDPP, GCN and Vegran, were based on HER2-postive patients. HDPP defined groups with better and worse prognosis, while GCN and Vegran were predictive of trastuzumab response.

The gene numbers of each signature reported in the original publications are shown in Table 1. Nine signatures (29%) consisted of less than 10 genes, 11 signatures (33%) 10~ 50, seven signatures (21%) 50~ 100, and six (18%) more than 100 genes. We could not collect the full gene lists for 2 signatures (less than100% coverage), because they contained some gene symbols that are unknown and some were duplicated in the same signatures.

### Common genes among the 33 signatures

A total of 2239 genes were obtained from the 33 signatures. The majority (1657) of these genes appeared in the signatures only once (Additional file 2: Table S2 and Additional file 3: Table S3). Two hundred thirty-eight genes were replicated: 173 genes were found in two signatures, 41 genes were found in three signatures, 14 genes were found in four signatures, 6 genes were found in five signatures, 4 genes were found in 6~ 8 signatures. There are a total 1895 unique signature genes after we removed replicated genes from the total 2239 genes (Additional file 8: Figure S1a).

**Fig. 2:**
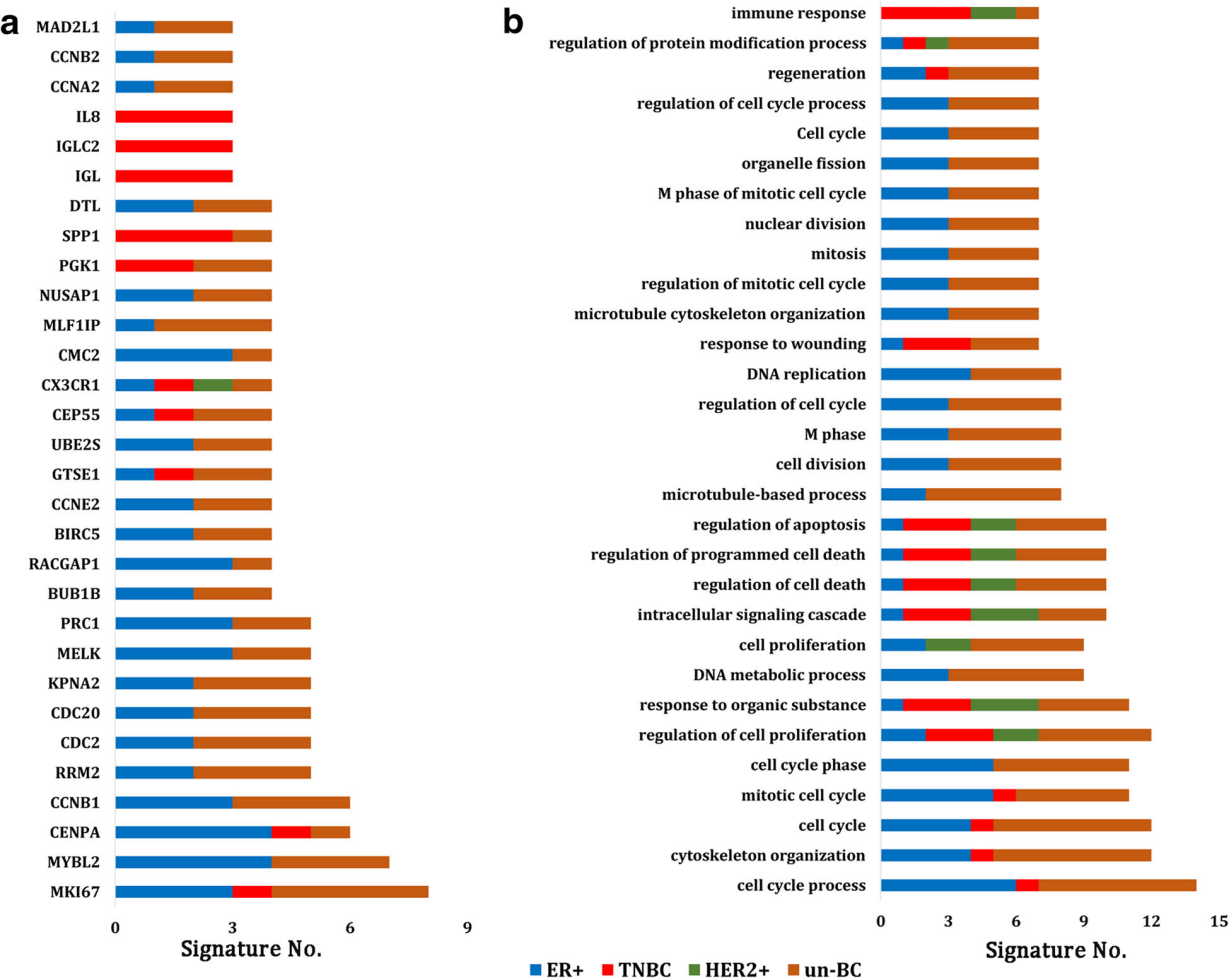
The common genes or function terms from breast cancer multiple signatures. **a** The top 30 common genes overlapped by different signatures. The official gene symbols were used for overlap analysis. **b** The top 30 common function terms among the 33 signatures. The GO biological process terms and KEGG pathways were analysed for each signature gene list using DAVID software. The signatures were grouped by tumour subtypes they were derived or applied for

We have ranked the individual signature genes based on their frequency of appearing in different signatures (Additional file 3: Table S3). The top 30 genes were shown in Fig. 2a. The top gene MKI67 (Marker of proliferation Ki-67) was found in eight signatures (CCPS, GGI97, IHC4, MBC, PAM50, Pawitan, Robust and RS) (Additional file 2: Table S2 and Additional file 3: Table S3). This gene encodes a nuclear protein that is associated with and may be necessary for cellular proliferation. However, except for MBC, the other 7 signatures were mainly based on ER-positive tumours or breast cancers which mostly included ER-positive patients. Most of these top ranked genes, such as MYBL2, CCNB1, RRM2, PRC1, MELK, KPNA2, CDC20, CDC2 and UBE2S, overlapped in signatures developed for ER-positive and mixed breast cancer subtypes. CX3CR1 was involved in four signatures that were grouped to different tumour subtypes.

The gene PGK1 was found in four signatures, and three out of the four signatures were derived from TNBC or invasive tumours. SPP1 was found in four signatures that were derived from ER-negative or TNBC or metastatic cancers.

**Fig. 3:**
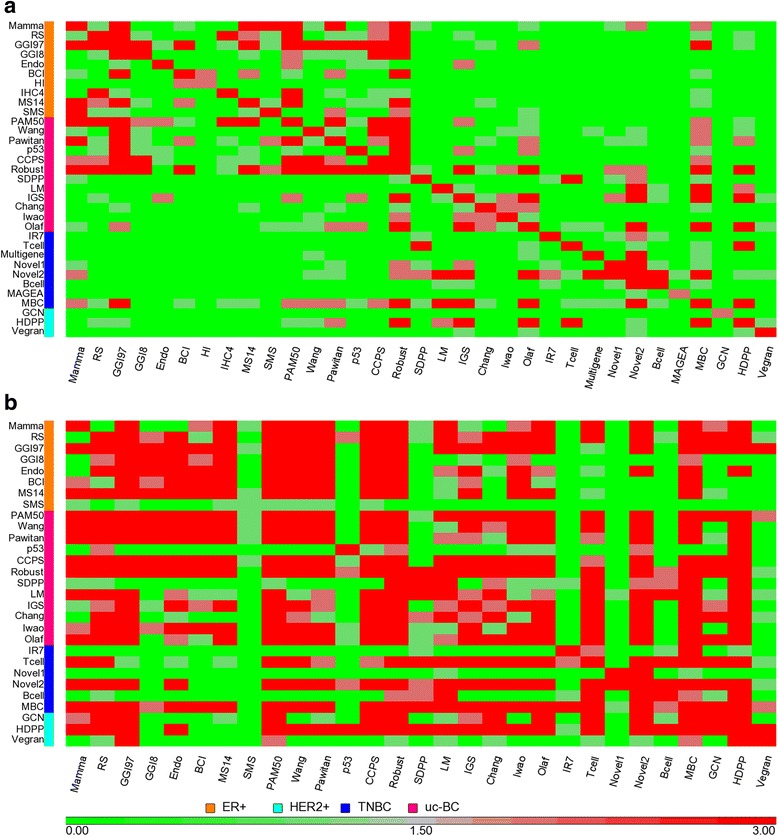
Heat map of the number of common genes or function terms between signatures. **a** A common gene matrix of 33 signatures by 33 signatures was shown by colour code. The two signatures share at least three common genes (red); the two signatures share one or two common genes (grey); the two signatures share none common genes (green). **b** A common function terms matrix of 29 signatures by 29 signatures was shown by colour code. Four out of the total 33 signatures did not have enriched significant function terms. The two signatures share at least three common terms (red); the two signatures share one or two common terms (grey); the two signatures share none common terms (green)

The number of overlapping genes among signatures is showed in Fig. 3a. It can be seen that there are two major areas in the heat map with more common genes than other areas. One has more red spots including signatures used for ER-positive breast cancers. Another contains signatures that were more relevant to ER-negative breast tumours. It might be expected that signatures developed from similar breast cancer cohorts in terms of known biology and developed for a known application would tend to have more common genes, but consistent with the also recognized heterogeneity in existing biological groups would not have 100% of genes in common. The two signatures, GGI97 and Wang, derived mostly from ER-positive patients using the same Affymetrix platform showed little overlap genes (Additional file 9: Figure S2).

### Common functions among the 33 signatures

A total of 1979 significant function terms were derived from enrichment analysis of each of the 33 signature gene lists (Additional file 5: Table S5) and 988 unique terms were obtained after removing replicates among signatures. The number of significant function terms of each signature generated by DAVID is listed in Additional file 1: Table S8. No significantly enriched function terms were found for four signatures (HI, IHC4, Multigene and MAGEA). There are 195 terms shared by any two signatures, 99 terms shared by three signatures, 58 terms shared by four signatures, 39 terms shared by five signatures, nine terms shared by six signatures, and 29 terms shared by seven or more signatures (Additional file 8: Figure S1b and Additional file 6: Table S6). The function terms were ranked by the frequency with which they appear in multiple signatures (Additional file 6: Table S6). The top 30 functional groups were shown in Fig. 2b and they are cell cycle, cell death, response to wounding, response to organic substance and intracellular signaling cascade.

The number of overlapping function terms among signatures is displayed in Fig. 3b. When we compared the heat map of function term frequency with that of gene frequency (Fig. 3a), it is evident that there are more common functions than common genes among these signatures. A similar situation was found when we examined the common genes and common functional groups within each subtypes (Additional file 10: Figure S3). Many signatures share at least two common function terms with each other even when they have zero overlapping genes. For example, there are no overlapping genes but at least two overlapping function terms between Mamma and several ER-negative breast cancer derived signatures (Tcell, HDPP, GCN). RS shared at least one common function term but zero overlapping genes with LM, Tcell, Novel2, Bcell, GCN, Vegran, Chang, Iwao and Olaf. Most of the latter signatures, have different applications compared to RS.

### Clustering 62 common genes on METABRIC dataset

**Fig. 4:**
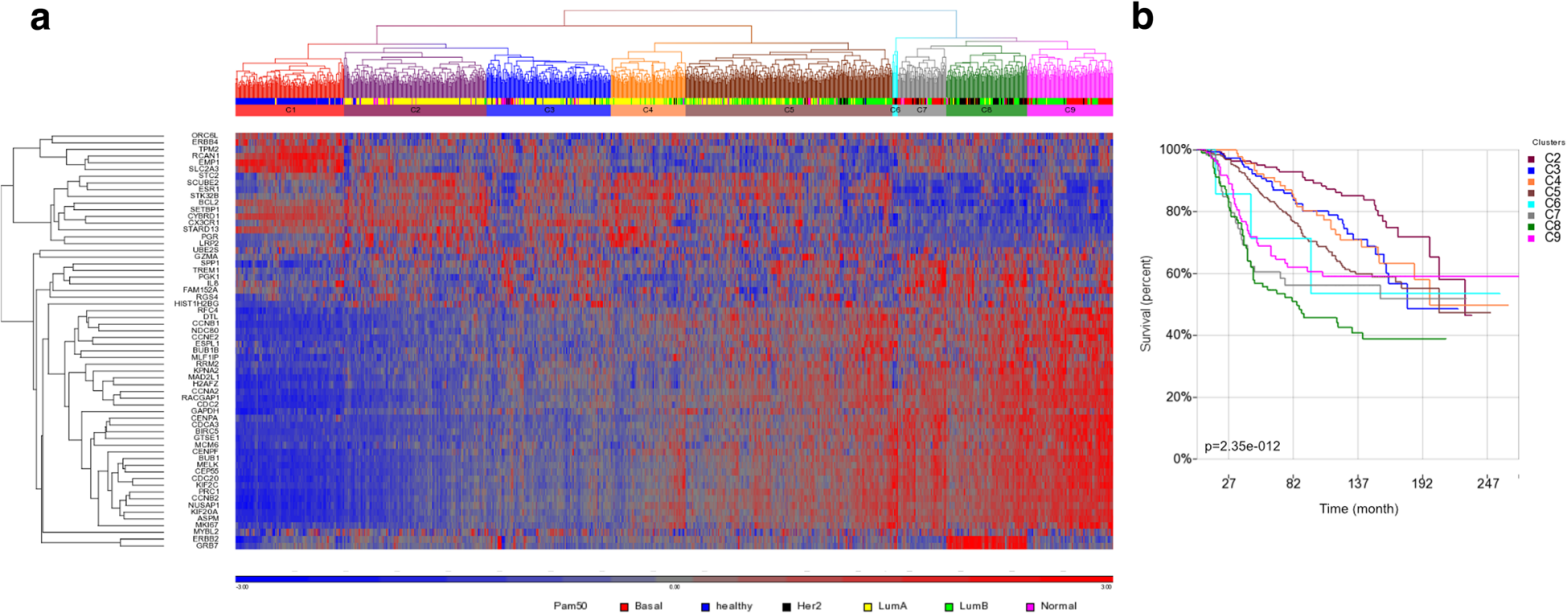
Evaluation of the value of the common genes in building breast cancer subtyping classifier. **a** 1141 METABRIC samples including 144 normal breast tissue samples were clustered by the 62 common genes that shared by at least three signatures using 2D Euclidean clustering with complete linkage settings. The clusters were selected by level 3 or 4 branches. Nine clusters were selected (C1 to C9) including the normal sample cluster C1. The PAM50 subtypes were indicated under the nine cluster colour bars. **b** Kaplan-Meier survival analysis for the nine subgroups. The disease specific survival (DSS) time was used for outcome endpoint

Sixty-two genes out of the 65 common genes in at least three signatures were found in the METABRIC data set and used for classifier testing. This analysis resulted in nine subgroups (Fig. 4a). As expected, the normal breast tissue samples were clustered in one group C1. Basal tumours were distributed in C7 and C9. Her2-enriched patients were in C8. The eight subgroups (not including the normal sample group) showed different clinical outcomes (Fig. 4b). The C8 had the lowest 10-year overall survival time while the C2 had the best overall survival time compared to all other clusters. The 62 genes presented two high-level clusters (upper branch, lower branch, left panel of Fig. 4a). The patients who had higher expression of upper branch genes tended to have worse outcome while the patients who had higher expression of lower branch genes tended to have better prognosis.

The upper branch of genes were most frequently found in signatures for ER-positive breast cancer or mixed subtypes and cell proliferation related, such as RFC4 (included in Mamma, MS14 and SMS) and KPNA2 (included in CCPS, GGI8, GGI97, Robust and Wang).

### Validation of the YMR model

**Fig. 5:**
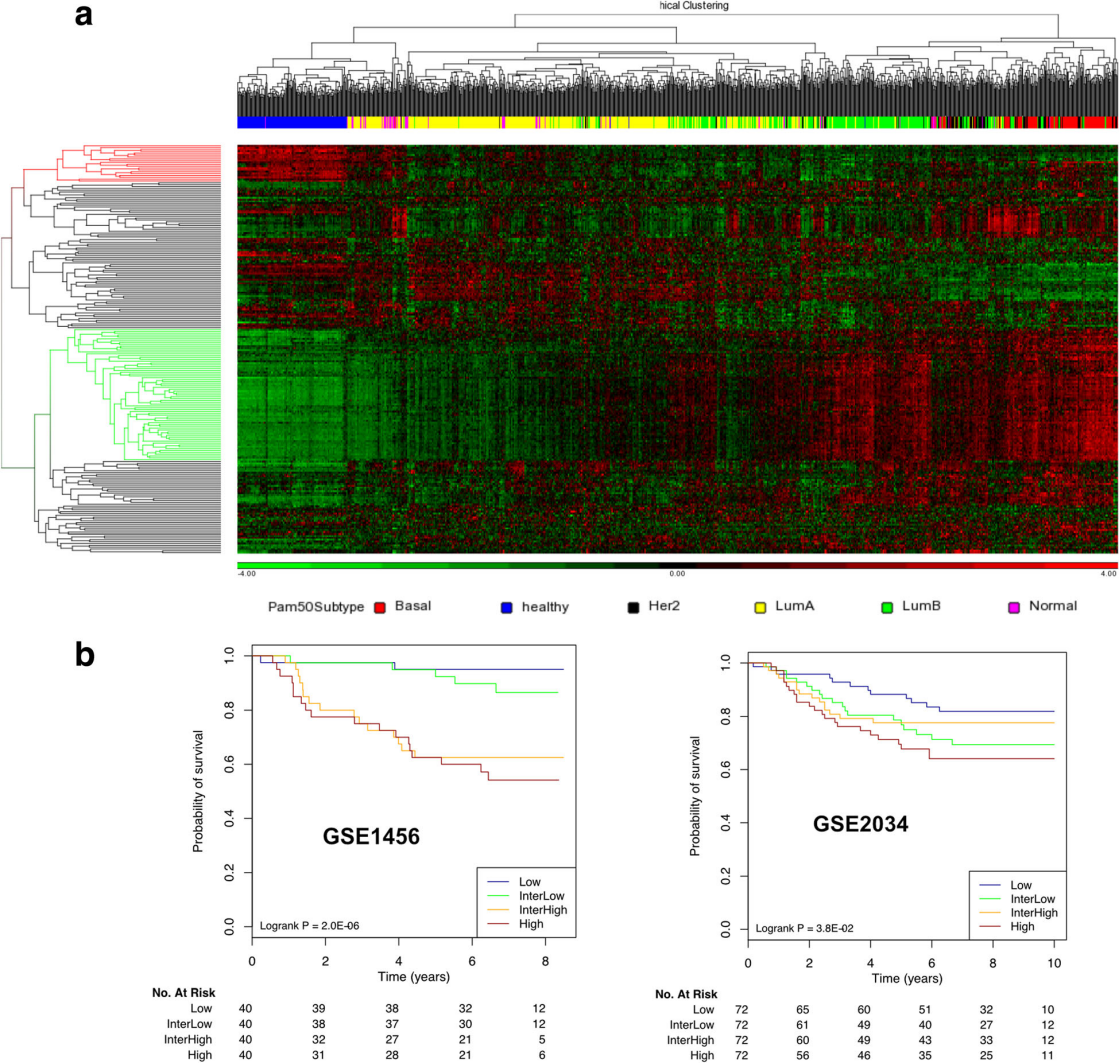
Evaluation of the Yin Yang gene expression ratio (YMR) model using the common genes. **a** Defining the Yin and Yang genes from the 220 common genes using the 1141 METABRIC samples data. The rows are the 220 genes that were shared by at least two signatures and the columns are the 1141 samples. The genes that showed consistently higher expression in normal breast tissue samples (in blue) and relative consistently lower expression in various tumour subtypes were selected as Yang genes (red). The genes that showed consistently lower expression in normal samples and higher expression in tumour samples were selected as Yin genes (green). The PAM50 subtypes were indicated by colour bars. **b** The YMR-all signature model developed by the selected Yin and Yang genes was tested by Kaplan-Meier survival analysis using two data sets

Based on the gene expression levels in tumour and normal breast tissue samples of the METABRIC data (Fig. 5a), we identified Yin and Yang genes from 220 of the 238 common genes that overlapped in at least two signatures and whose expression value could be extracted from METABRIC. Twenty genes (gene cluster in red) which had higher expression level in normal (healthy) than in tumours (Lum A, Lum B, Her2, Normal and Basal) were selected as Yang genes. Seventy-one genes (gene cluster in green) had higher expression level in tumours than in normal were used as Yin genes. The 71 Yin genes were enriched with cell cycle and 20 Yang genes were involved in diverse functions, such as secreted extracellular region, signal peptide, disulfide bond, regulation of apoptosis and blood circulation (Additional file 11: Table S9). The YMR model was built from all subtypes (YMR-all) using the ratio of the mean of all the Yin genes expression and the mean of all the Yang genes expression. Patients were then divided into four groups with low, inter-low, inter-high, high risk of death according to YMR scores. As shown in Fig. 5b, the YMR-all can stratify patients into four risk groups using different patient cohorts and data sets, with data set GSE1456 showing more significance than GSE2034 data set. The low risk patients in the two datasets had significantly higher 10-year overall survival probability than the high risk patients.

### Comparing signatures

**Fig. 6:**
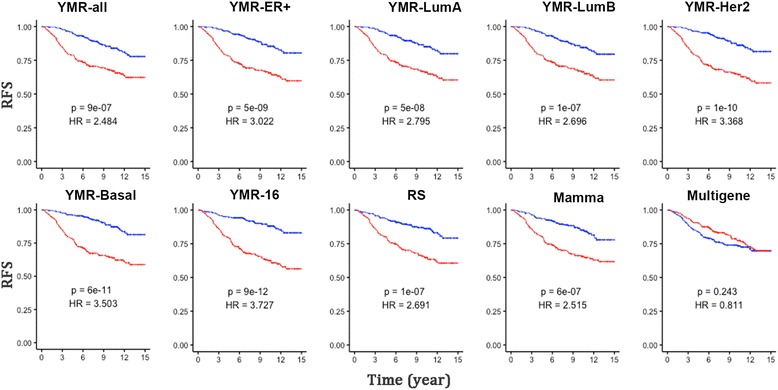
Signature comparison in ER+/Node-negative patients. The comparison was conducted bioinconductor package geneFu using patient samples from 5 Bioconductor data sets (Materials and Methods). Total 541 ER+/Node- patients who did not undertake adjuvant treatment were stratified by the median score of each signature and the significance was assessed by log-rank test of the Kaplan-Meier analysis using the recurrence free survival (RFS) rate

We compared the YMR-all model with MammaPrint (Mamma), OncotypeDx (RS) and Multigene HRneg/Tneg signature (Multigene) and our previously reported 16-gene YMR (YMR-16). There are two genes (CDC20 and UBE2C) and three GO terms (cell division, Oocyte meiosis, and cell cycle) overlapped between these two YMR models. We examined if these signatures were able to stratify patients within each subtype of ER-positive/node-negative, luminal A, luminal B, HER2-enriched and basal breast cancers. For early stage ER+/Node-negative, the YMR model showed significance (HR = 2.5, *p* = 9e − 07), but a little less significance than MammaPrint (HR = 2.52, *p* = 6e-07), OncotypeDx (HR = 2.7, *p* = 1e-07), and YMR-16 (HR = 3.7, *p* = 9e-12) (Fig. 6). Only signature Multigene did not show significance in stratifying ER-positive/node-negative patients (HR = 0.81, *p* = 0.24) but worked for basal subtype (HR = 2, *p* = 0.04, Additional file 12: Figure S4) since it was developed from TNBC [31]. In LumA subtype, only OncotypeDx and YMR-16 showed significant stratification (Additional file 12: Figure S4). MammaPrint, OncotypeDx, YMR-16 can stratify lumB significantly.

It is noteworthy that although the YMR-all model significantly stratified ER+/Node- patients, it is not superior to the currently used signatures RS and Mamma. One explanation is YMR-all was selected from clustering and tested without optimization. The other could be that the YMR-all was derived from a heterogeneous cohort containing all subtypes. Therefore we selected YMR genes from different subtypes by clustering (Additional file 13: Figure S5A, B, C, D, E), and tested their performances in stratifying the same subtype cohorts. As expected, the YMR_ER+ derived from ER+/Node- patients had superior performance than either RS or Mamma on ER+/Node- data (Fig. 6). However this approach did not work for other subtypes. Interestingly, we found various YMR signatures intended to work for Her2 subtype but with an opposite direction (Additional file 12: Figure S4). Since the current study focuses on the questions of functions and pathways of previously reported signatures, further study will be needed to look into these findings and optimising their performances.

## Discussion

The finding that there is little overlap of constituent genes amongst molecular signatures generated by different researchers using different patient cohort data has long been a source of concern and the reasons for it are still the focus of ongoing debate [49]. Several explanations have been proposed [24]. One is that researchers have used different platform technologies and supervised protocols for signatures derivation. Another focuses on the heterogeneity of samples included in the different datasets. Small sample size is also another possible contributor to the differences seen [24]. However, it is possible that a group of distinct genes actually support the same function, and there are limited studies that focus on such an analysis of the genes within different signatures [24].

Breast cancer is well known to be heterogeneous. The differences in clinical composition of the existing different datasets may partly explain the little overlap already found amongst different signatures. However even the number of overlapping genes within the known ER-positive/negative subgroups is still small^21^ and the one-size-fits-all signature will not be possible. In our study, signatures for the currently defined same type of breast cancers (ER-positive/negative), tended to have more common genes with each other than signatures for different breast cancer subtypes (Fig. 3a). However, there are still known and unknown forms of heterogeneity that exist among the same ER status subtypes that could partly contribute to the limited gene overlap problem.

Many gene signatures were developed by selecting genes whose expression levels correlate to the clinical outcomes without any focus on gene functions. We hypothesize that the genes in these reported signatures would functionally associate to the diseases, either directly or indirectly. Thirty-three signatures were included in this study. Two hundred thirty-eight of the total 2239 genes were shared by at least two signatures. However, 429 of the total 1979 function terms derived from the signatures were common in at least two signatures. These function terms were cell cycle, cell death, response to wounding, response to organic substance and intracellular signaling cascade. The fact that signatures shared more function terms than genes supports our hypothesis that the signature genes represent similar functions or pathways despite actual different individual genes.

In this study we found that most of the signatures from ER-positive breast cancers had common function terms focused on cell proliferation although they did not share common genes. Functions impacting cell proliferation, such as cell cycle process, mitotic cell cycle, DNA replication, nuclear division were shared by signatures mostly used for ER-positive, such as Mamma, RS, Endo, and GGI97. Most of the signatures from ER-negative tumours shared common function terms focused on immune response though they did not share common genes. Interestingly, we found the signatures derived from ER-negative tumours, such as Novel1, Novel2, MBC, shared common functions associated with immune response including lymphocyte activation, leukocyte activation and T cell activation. It has been reported that activation of complement and immune response pathways are associated with good prognosis in a subclass of basal tumors [18].

One point worth noting is that there were also common function terms between the signatures from ER-positive and ER-negative breast cancers including cell death, regulation of cell proliferation, response to organic substance, intracellular signaling cascade, response to hormone stimulus, response to oxygen levels, bone development, DNA packaging, response to hypoxia, ossification and skeletal system development. It indicates that the genes of these function terms are generally associated with prognosis and treatment response prediction in different breast cancer types. This implies that in tumour progression, different subtypes undergo similar biological processes. The same ER+ prognosis using the same platform (qRT-PCR), signature Endo shares the top pathway regulation of cell cycle process (GO: 0010564) with signature MS14 while this pathway shares zero genes between these two signatures, again consistent with the concept that the pathway is more important than the actual genes. Our study strongly implies that the prognosis of different subtypes may be determined by the similar biological processes or pathways. However, different subtypes may have specific pathways that can be added to or impact on the common pathways, i.e. ER or HER2. For example, signature OncotypeDx was developed for ER+, signature MBC is for TNBC, the pathway response to wounding (GO: 0009611) is common, but pathway leukocyte activation (GO: 0045321) is unique for signature MBC. It was previously reported that breast cancer patients whose tumors expressed wound-response genes had significantly poorer outcomes in both overall survival and distant metastasis-free survival than tumors that did not express wound-response genes [5].

In addition to proliferation pathways, we also find cytoskeleton organization pathways are common among ER+ signatures (MammaPrint, 97-gene GGI, BCI, MS14) as well as organelle fission pathways are common to 97-gene GGI, BCI, MS14. Common functions were found for signatures for different subtypes. MammaPrint was used for ER+ and shared no common genes with several ER-negative breast cancer derived signatures (HDPP, Tcell, and GCN) but shared pathway intracellular signalling cascade (GO: 0007242), pathways in cancer (hsa05200), response to oxygen levels (GO:0070482) with HDPP, shared pathway positive regulation of signal transduction (GO:0009967) with Tcell. We also found functions, such as regulation of protein modification, regulation of cell death, are enriched in signatures used for different subtypes (ER+, TNBC, HER2+).

Another argument is that a statistical association between multi-gene signatures and clinical outcomes does not necessarily imply biological significance [50]. For example, Miller and colleagues [7] developed a 32-gene expression signature which indicated p53 status (mutant and wild-type). However, none of the 32 genes were known transcriptional targets of p53 or known to be involved in the p53 pathway. A potential explanation could be that most of these signatures were identified using Cox regression, which simply selected the top-ranked genes using a Cox score [51]. In this study, we hypothesized that two opposing effects called Yin and Yang determine the fate of tumour cells. We used Yin to represent the effects leading to cancer progression and Yang as the effects to maintain the normal healthy status. In this context, we tried to develop signatures that could indicate the biological mechanisms in breast cancer progression.

Interestingly, many signature genes do not show a difference in expression level between tumour and normal breast tissue samples, at least at the RNA level. We selected 71 Yin genes and 20 Yang genes from the signature genes. Functional annotation showed that most of the Yin genes functioned in the cell cycle, while the enriched function terms of the 20 Yang genes were more diverse such as secreted extracellular region, signal peptide, disulfide bond, regulation of apoptosis and blood circulation. In this study we used GO biological process terms and KEGG pathways which may be different from those that were used in other studies focused on function terms and pathways.

Urgent work is needed for personalized care for TNBC because TNBC is more heterogeneous and more aggressive than ER-positive breast cancers. Current signatures developed for TNBC have not been used clinically mostly due to the lack of further validation and/or poor reproducibility. We found that five signatures were derived from TNBC (Multigene, Bcell, Novel1, Novel2 and MAGEA), one from basal tumours (MBC), two from ER-negative (IR7 and Tcell), and three from invasive/metastatic/tumorigenic breast cancers (SDPP, LM and IGS). Interestingly, these signatures shared more common genes with each other than they shared with others derived from ER+ and/or Her2 enriched subtypes. We selected 64 genes common to at least two of these 11 signatures to classify 127 TNBC patients from METABRIC dataset (Additional file 14: Figure S6) and 107 TNBC patients from GSE58812 dataset (Additional file 15: Figure S7). Two clusters were identified in both the two datasets. Most of these common genes had a high expression in one cluster while a low expression in another cluster. The cluster with higher expression had a better overall survival rate however with a modest significance (*p* = 0.14 for METABRIC, *p* = 0.13 for GSE58812). This inferred that the expression level of these genes could be associated with the progression of the aggressive breast cancers. The top pathway enriched in the 64 genes from these 11 signatures was “regulation of immune response” (*p* = 1.8E-4, FDR = 8.5E-2).

One of the limitations of this study is we used the same significant *p*-value as the cutoff to evaluate the functional groups of signatures. This informs what functional group a signature may be involve in, but does not tell the significance. However, it is challenging to compare the significance of signatures with different gene list size. The second limitation is we lacked the optimization and large dataset validation using common genes for YMR signature model development, though this is not the focus of this study.

## Conclusions

In summary, the long debate about the underlying reasons for the lack of gene overlap in multi-gene signatures derived from different research studies can be partially resolved by our discovery that often the signature genes are associated with similar functions and/or pathways despite being distinct individual genes. Though diverse types of signatures with different gene constituents are expected due to the heterogeneity of breast cancer, the gene signatures for the same subtypes of breast cancers would be expected to participate in similar functions. The genes collected from these previously reported signatures have also proved to be a valuable resource for new model development.

## Additional files

Additional file 1: **Table S1.** Signatures summary. A summary of 33 signatures about the platforms derived from, the subtypes used for, the gene number included, and the function terms involved. **Table S8.** The number of function terms in each signature. (PDF 82 kb)

Additional file 2: **Table S2.** Gene distribution among the signatures. A matrix with each of all signature genes against each of the 33 signatures to see how the genes distribute among those signatures. (CSV 337 kb)

Additional file 3: **Table S3.** The number of signatures involved in each gene. In this table, for each signature gene, the number of signatures including this gene was counted. (CSV 29 kb)

Additional file 4: **Table S4.** The number of common genes between every two signatures. A matrix of 33 signatures by 33 signatures with the number of common genes shared by every two signatures. (CSV 4 kb)

Additional file 5: **Table S5.** Function terms distribution among the signatures. A matrix with each of all signature genes against each of the 29 signatures to see how the function terms distribute among those signatures. Since no significantly enriched function terms were found for four signatures (HI, IHC4, Multigene and MAGEA), 29 of the 33 signatures were used for analysis. (CSV 194 kb)

Additional file 6: **Table S6.** The number of signatures involved in each function term. In this table, for each signature function term, the number of signatures including this gene was counted. (CSV 57 kb)

Additional file 7: **Table S7.** The number of common function terms between every two signatures. A matrix of 29 signatures by 29 signatures with the number of common function terms shared by every two signatures. No significantly enriched function terms were found for four signatures (HI, IHC4, Multigene and MAGEA), 29 of the 33 signatures were used for analysis. (CSV 3 kb)

Additional file 8: **Figure S1.** Genes and function terms among signatures. A. Common genes discovered in signatures (upper panel). Gene number in more than certain number of signatures were indicated. The unique genes were counted in each portion (bottom panel). B. Common function terms enriched in signatures (upper panel). Term number in more than certain number of signatures were indicated. The unique terms were counted in each portion (bottom panel). (PDF 77 kb)

Additional file 9: **Figure S2.** Venn diagram of common genes number in ER-positive signatures from different platform. Common genes among signatures derived from several different platforms but used for ER-positive patients or mixed subtypes. (TIFF 817 kb)

Additional file 10: **Figure S3.** Heat map of the number of common genes or function terms between signatures in four subgroups. The number of common genes or function terms among signatures in the four subgroups (ER+, HER2+, TNBC, uc-BC) were compared. The two signatures sharing at least three common genes or function terms present red; the two signatures share one or two common genes/terms present grey; the two signatures share none common genes or terms present green. (TIFF 572 kb)

Additional file 11: **Table S9.** The selected Yin Yang gene lists with its enriched function terms. We showed the Yin gene list and the significant function terms of this gene list generated by DAVID in this table. We also showed the Yang gene list and the significant function terms of this gene list generated by DAVID. GO term Biological process and KEGG pathway were used to define the functions enrichment with the default settings (EASE *p*-value less than 0.1). (XLS 13 kb)

Additional file 12: **Figure S4.** Signatures Comparison in different subtypes. YMR models were compared with MammaPrint (Mamma), OncotypeDx (RS) and the Multigene HRneg/Tneg signature (Multigene) and the previously reported 16-gene YMR(YMR-16). Signatures were evaluated in stratifying within each of luminal A (LumA, *n* = 310), luminal B (LumB, *n* = 209), HER2-enriched (Her2, *n* = 87) and basal (Basal, *n* = 113) subtype breast cancers. Five datasets from Bioconductor libraries: breastCancerMAINZ (GSE11121), breastCancerTRANSBIG (GSE7390), breastCancerUPP (GSE3494), breastCancerUNT (GSE2990), breastCancerNKI, and the geneFu package were used for these comparisons. All patients did not undertake adjuvant treatment. Each cohort was stratified by the median score of each signature and the significance was assessed by log-rank test of the Kaplan-Meier analysis. (PDF 600 kb)

Additional file 13: **Figure S5.** Selection of Yin and Yang gens for different subtypes. The 220 common signature genes expression data of various cancer subtypes were extracted from METABRIC expression data set. The genes (rows) were clustering among each subtype (columns) and the normal samples (A, B, C, D, E). The contrast clusters were selected as Yin genes (in blue) and Yang (in red) genes. (PDF 1554 kb)

Additional file 14: **Figure S6.** Building TNBC subtyping classifier in METABRIC using common genes from TNBC signature group. Sixty-four genes overlapped in at least two of 11 signatures were used to classify 127 TNBC patients from METABRIC dataset into two clusters. Among these 11 signatures, 6 signatures (Multigene, Bcell, Novel1, Novel2, MBC and MAGEA) were derived from TNBC, two (IR7 and Tcell) from ER-negative patients, three (SDPP, LM and IGS) from a mixed subtype patients. The cluster with higher expression had a better overall survival rate however with a modest significance (*p* = 0.14). (TIFF 640 kb)

Additional file 15: **Figure S7.** Building TNBC subtyping classifier in GSE58812 using common genes from TNBC signature group. Six signatures (Multigene, Bcell, Novel1, Novel2, MBC and MAGEA) were derived from TNBC, two (IR7 and Tcell) from ER-negative patients, three (SDPP, LM and IGS) from a mixed subtype patients. These signature shared more common genes with each other than they shared with others. Thus genes of this 11 signatures were pooled together. Sixty-four genes overlapped in at least two of these 11 signatures were used to classify 107 TNBC patients from GSE58812 dataset into two clusters. The cluster with higher expression had a better overall survival rate however with a modest significance (*p* = 0.13). (TIFF 543 kb)

## Abbreviations

METABRIC: Molecular Taxonomy of Breast Cancer International Consortium
TNBC: triple-negative breast cancers
YMR: Yin Yang gene Mean expression Ratio
