# Supplementary material for: Genes and functions from breast cancer signatures
Source: BMC Cancer. 2018 Apr 27;18:473. doi: 10.1186/s12885-018-4388-4 (PMC5921990; doi:10.1186/s12885-018-4388-4)
Supplement: Supplementary file 1 — Table S1. Signatures summary. A summary of 33 signatures about the platforms derived from, the subtypes used for, the gene number included, and the function terms involved. Table S8. The number of function terms in each signature. (PDF 82 kb) [file 12885_2018_4388_MOESM1_ESM.pdf]

Table S1. Signatures summary.

A summary of 33 signatures about the platforms derived from, the subtypes used for, the gene number included, and the function terms involved.

classes		Signatures number (n)	
**Subtype used for**		**33**	
	ER-positive		10
	ER-negative		2
	HER2-enriched		3
	Triple negative/basal		6
	mix subtypes		12
**Platform derived from**		**33**	
	Affymetrix microarray		18
	Agilent microarray		6
	RT-PCR/qRT-PCR/ATAC-PCR		7
	- FISH		1
	Immunohistochemistry		1
	other microarray		2
**Number of genes included**		**33**	
	< 10		9
	10~50		11
	50~100		7
	> 100		6
**Number of function terms included**		**29**	
	< 10		5
	10~50		11
	50~100		5
	> 100		8

Table S8. The number of function terms in each signature.

signature_union	number_of_term
Tcell	199
HDPP	187
MBC	187
Robust	177
PAM50	163
Novel2	159
GGI97	143
CCPS	111
Olaf	94
Iwao	74
LM	65
Pawitan	59
RS	55
Chang	50
IGS	41
GCN	31
SDPP	31
Wang	27
Mamma	23
Endo	21
MS14	21
Bcell	12
BCI	12
Vegran	11
IR7	8
Novel1	7
p53	6
GGI8	4
SMS	1

The number of significant function terms of each signature generated by DAVID is listed in this table. We selected GO term Biological process and KEGG pathways to define the functions enrichment with the default settings (EASE p-value less than 0.1). No significantly enriched function terms were found for four signatures (HI, IHC4, Multigene and MAGEA).
