# Supplementary figures and images for: Genes and functions from breast cancer signatures
Source: BMC Cancer. 2018 Apr 27;18:473. doi: 10.1186/s12885-018-4388-4 (PMC5921990; doi:10.1186/s12885-018-4388-4)

**A**

Total gene No.

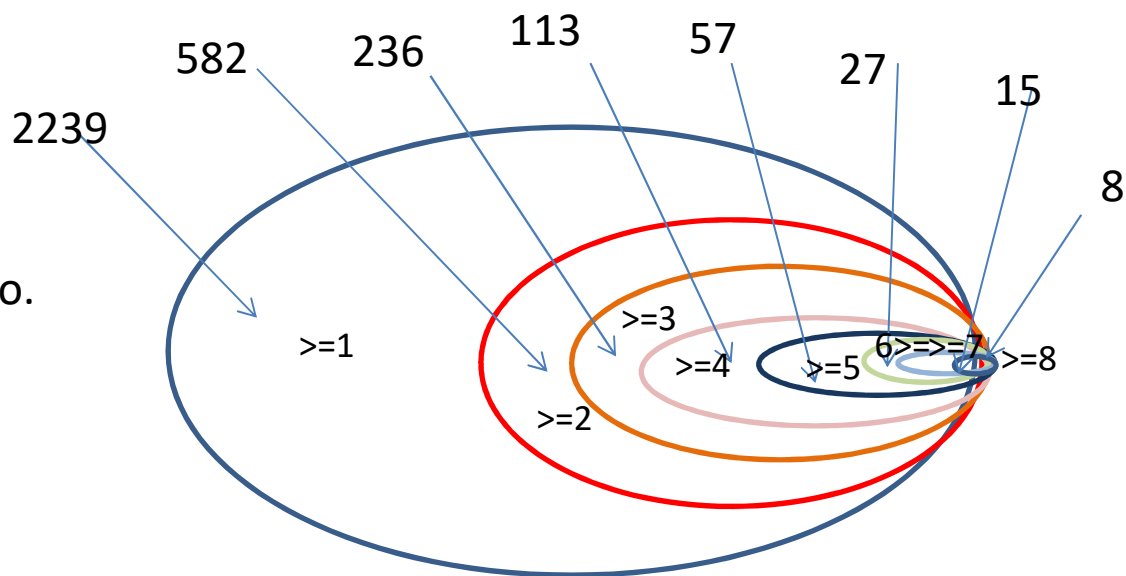

Unique gene No.

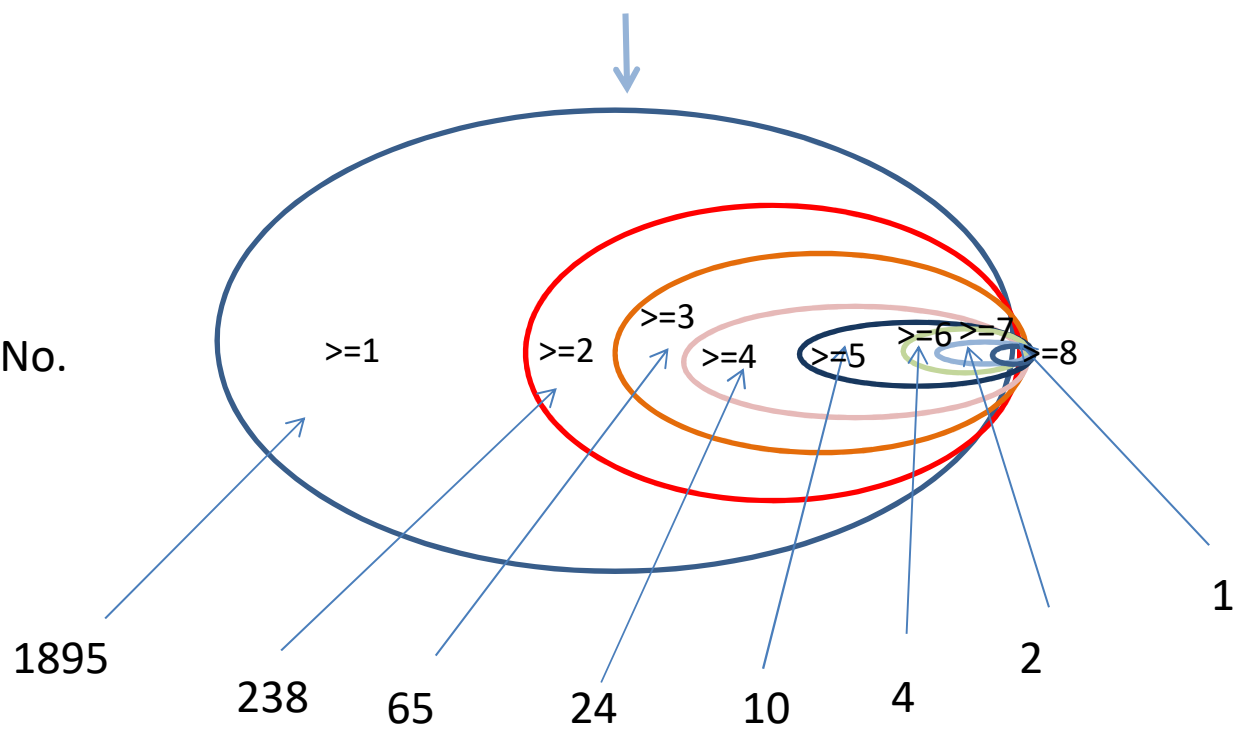

**B**

Total Function  
term No.

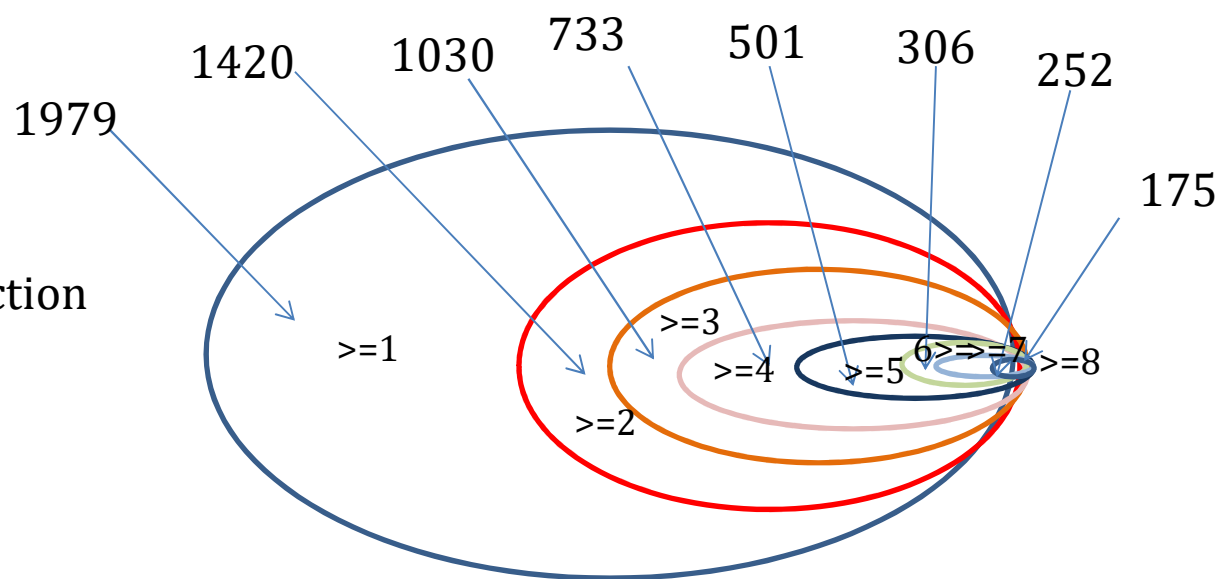

Unique function  
term No.

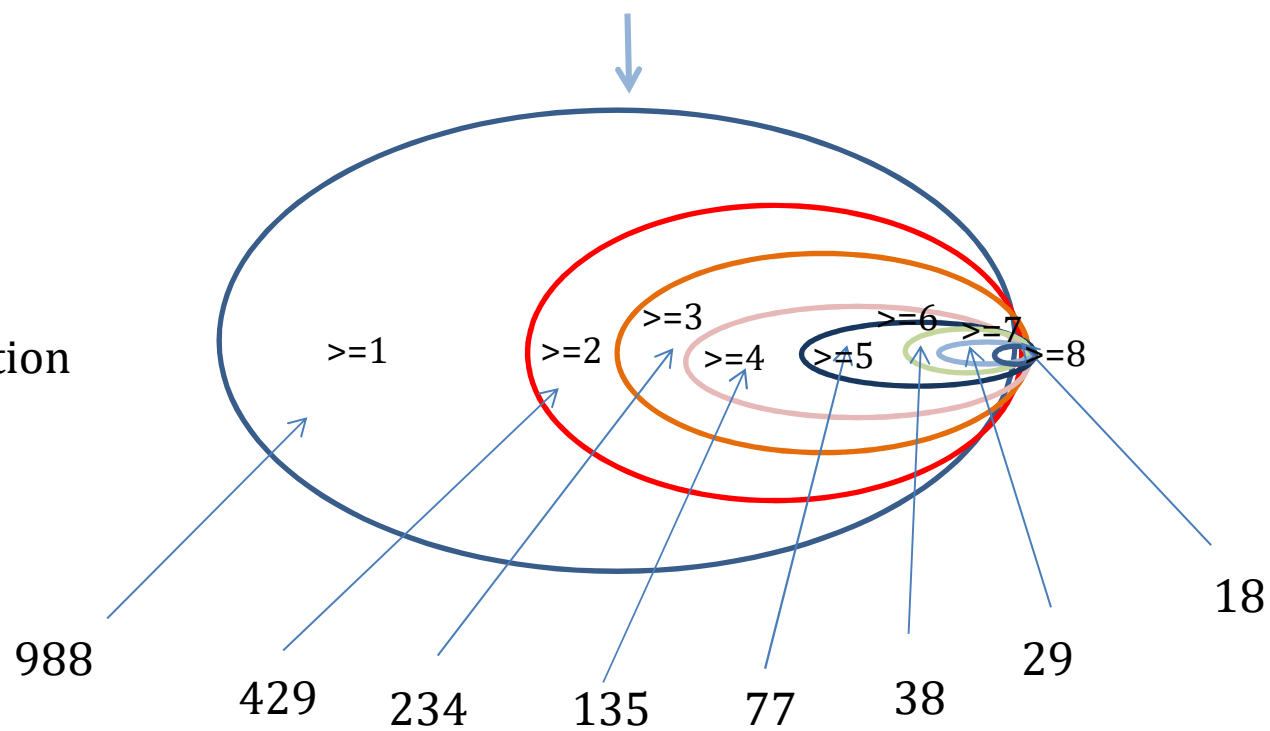

Supplement: Supplementary file 8 — Figure S1. Genes and function terms among signatures. A. Common genes discovered in signatures (upper panel). Gene number in more than certain number of signatures were indicated. The unique genes were counted in each portion (bottom panel). B. Common function terms enriched in signatures (upper panel). Term number in more than certain number of signatures were indicated. The unique terms were counted in each portion (bottom panel). (PDF 77 kb) [file 12885_2018_4388_MOESM8_ESM.pdf]

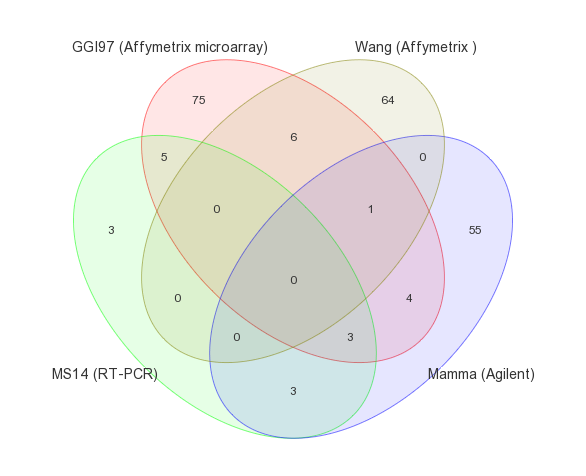

Supplement: Supplementary file 9 — Figure S2. Venn diagram of common genes number in ER-positive signatures from different platform. Common genes among signatures derived from several different platforms but used for ER-positive patients or mixed subtypes. (TIFF 817 kb) [file 12885_2018_4388_MOESM9_ESM.tiff]

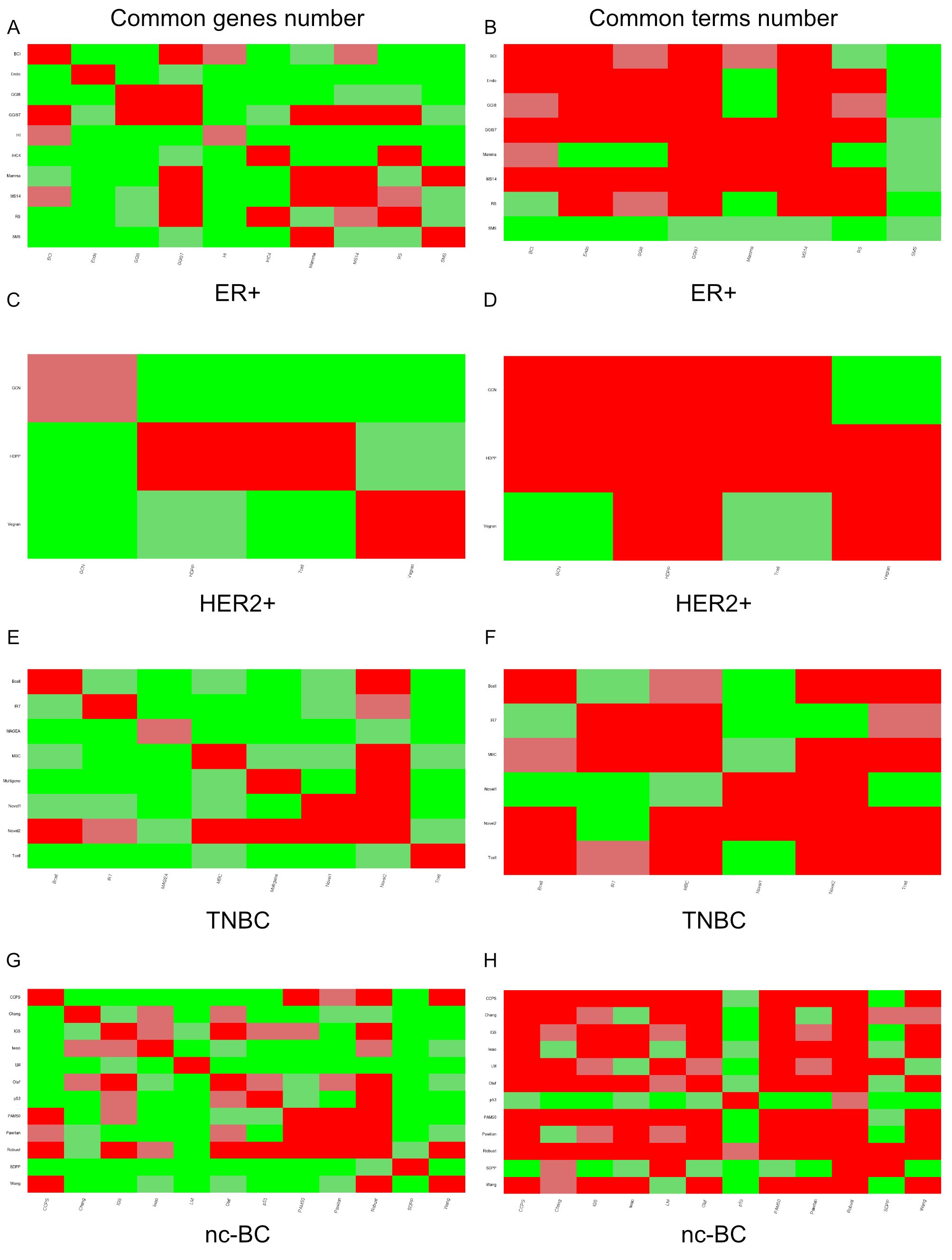

Supplement: Supplementary file 10 — Figure S3. Heat map of the number of common genes or function terms between signatures in four subgroups. The number of common genes or function terms among signatures in the four subgroups (ER+, HER2+, TNBC, uc-BC) were compared. The two signatures sharing at least three common genes or function terms present red; the two signatures share one or two common genes/terms present grey; the two signatures share none common genes or terms present green. (TIFF 572 kb) [file 12885_2018_4388_MOESM10_ESM.tiff]

YMR-all

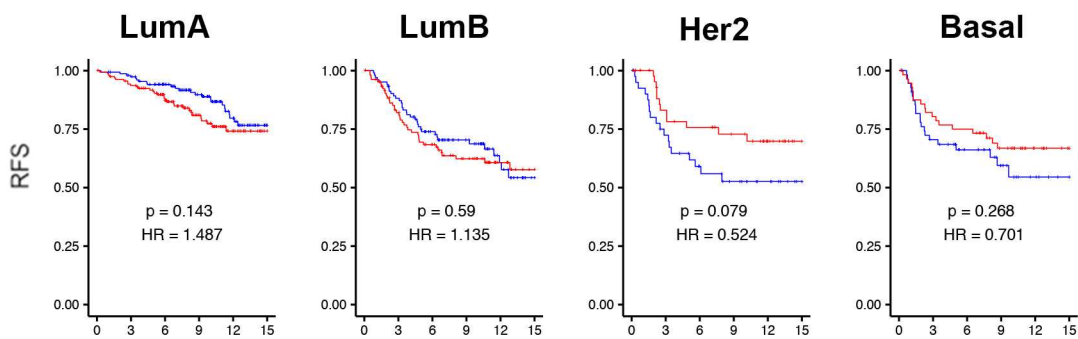

YMR-ER+

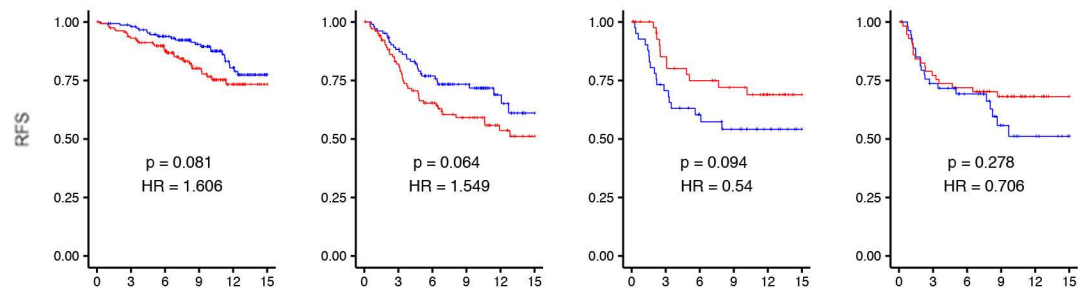

YMR-LumA

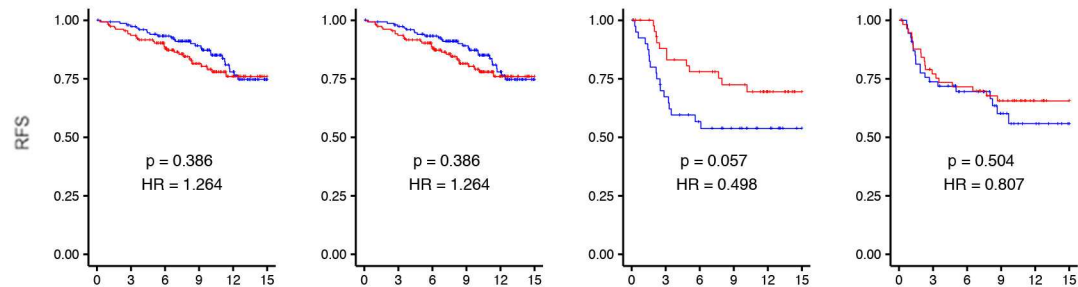

YMR-LumB

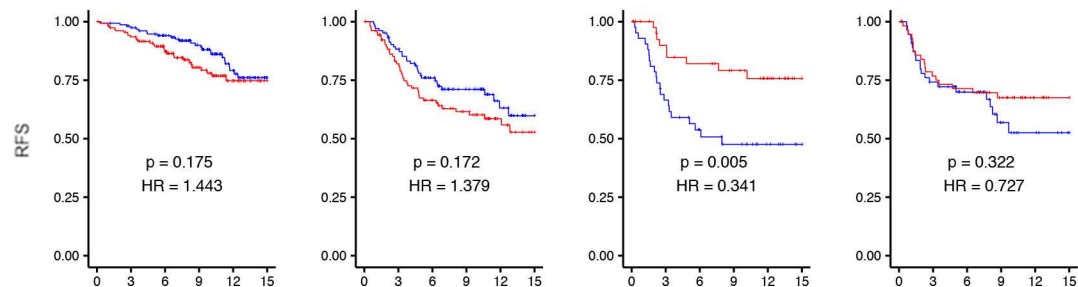

YMR-Her2

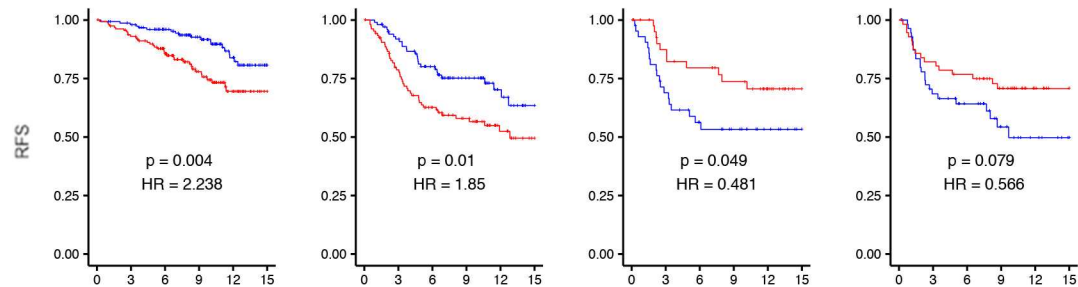

Time (year)

YMR-Basal

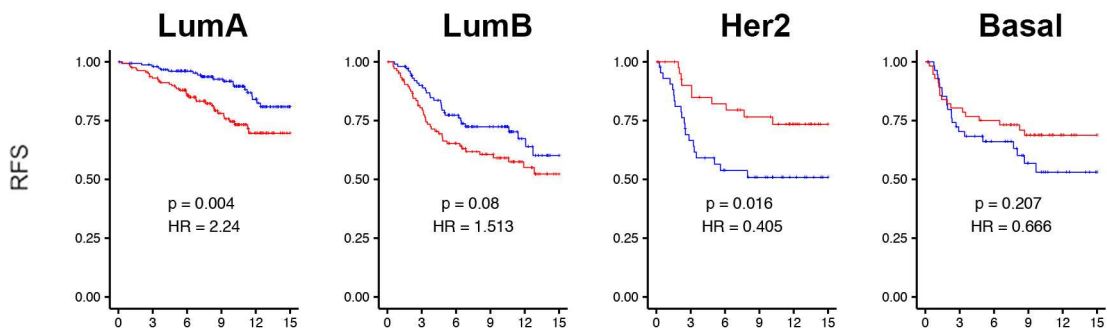

YMR-16

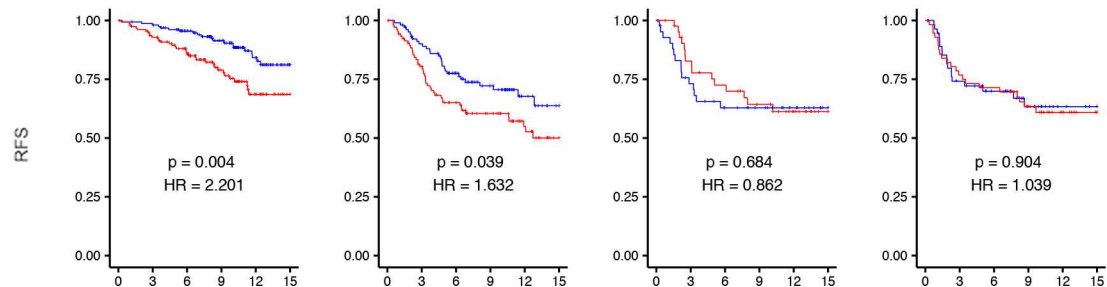

RS

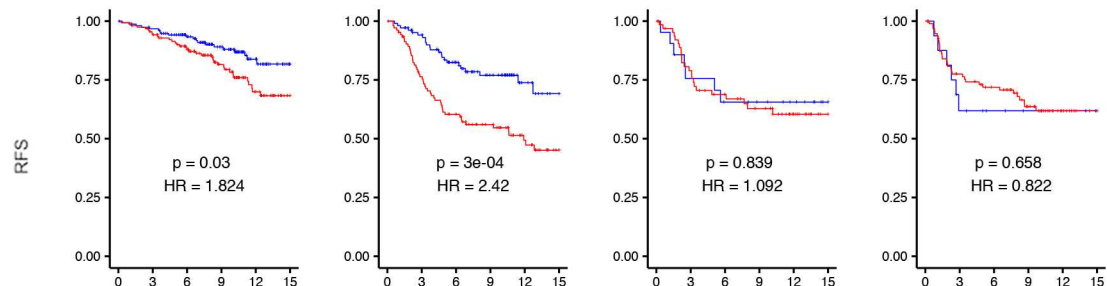

Mamma

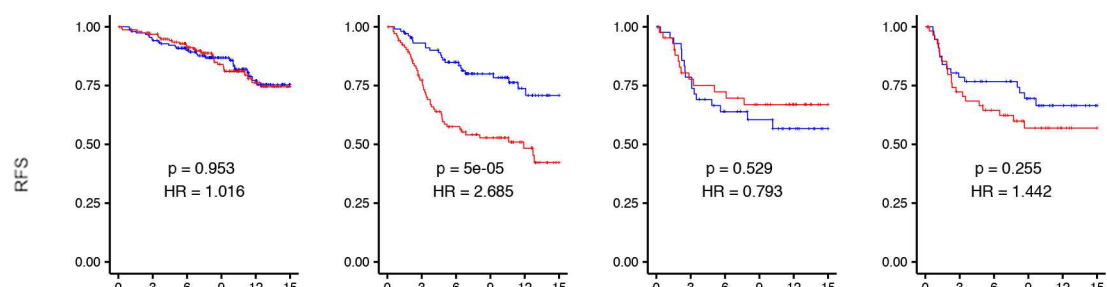

Multigene

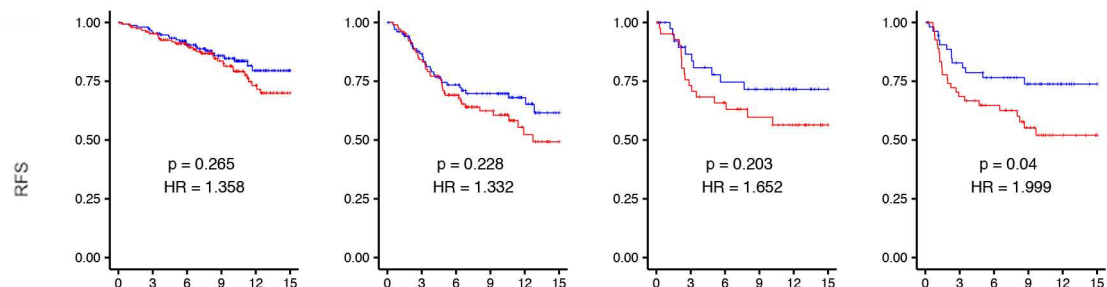

Time (year)

Supplement: Supplementary file 12 — Figure S4. Signatures Comparison in different subtypes. YMR models were compared with MammaPrint (Mamma), OncotypeDx (RS) and the Multigene HRneg/Tneg signature (Multigene) and the previously reported 16-gene YMR(YMR-16). Signatures were evaluated in stratifying within each of luminal A (LumA, n = 310), luminal B (LumB, n = 209), HER2-enriched (Her2, n = 87) and basal (Basal, n = 113) subtype breast cancers. Five datasets from Bioconductor libraries: breastCancerMAINZ (GSE11121), breastCancerTRANSBIG (GSE7390), breastCancerUPP (GSE3494), breastCancerUNT (GSE2990), breastCancerNKI, and the geneFu package were used for these comparisons. All patients did not undertake adjuvant treatment. Each cohort was stratified by the median score of each signature and the significance was assessed by log-rank test of the Kaplan-Meier analysis. (PDF 600 kb) [file 12885_2018_4388_MOESM12_ESM.pdf]

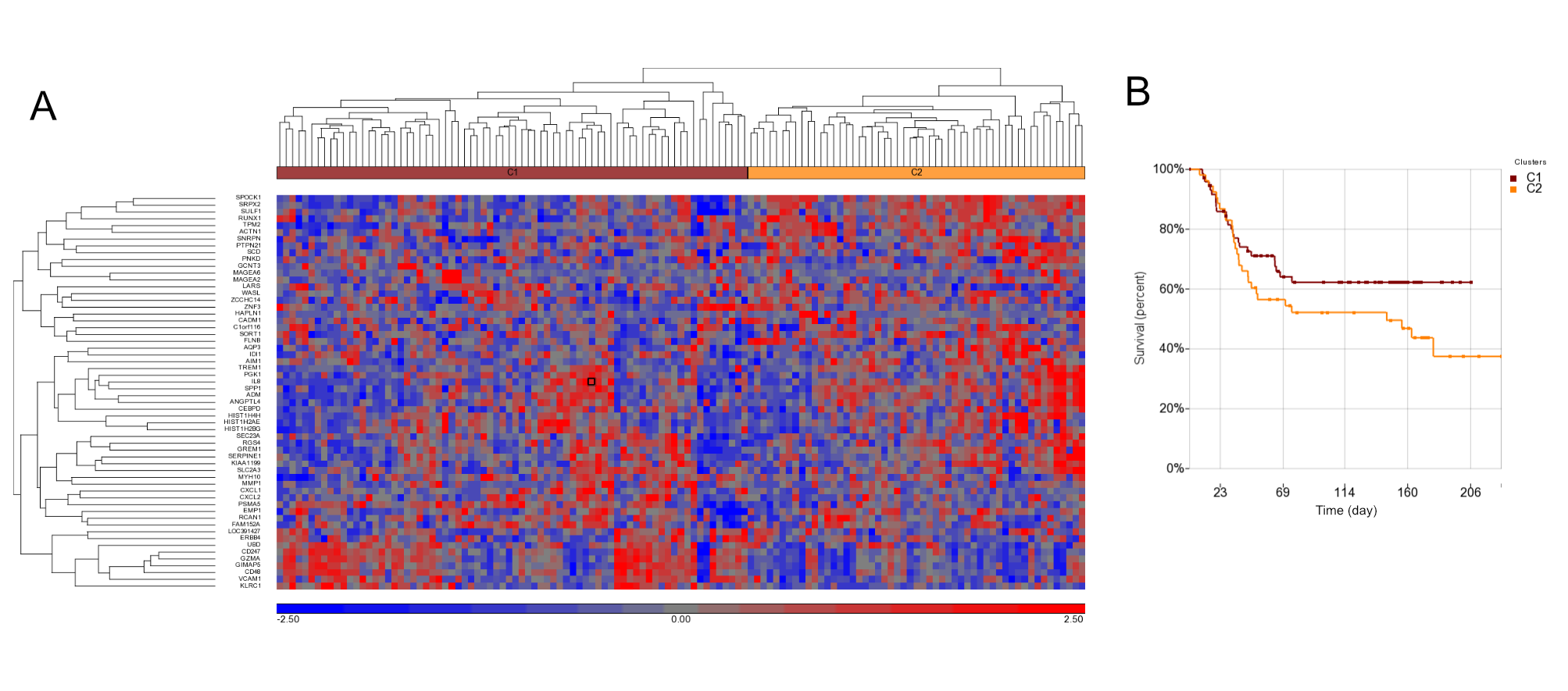

Supplement: Supplementary file 14 — Figure S6. Building TNBC subtyping classifier in METABRIC using common genes from TNBC signature group. Sixty-four genes overlapped in at least two of 11 signatures were used to classify 127 TNBC patients from METABRIC dataset into two clusters. Among these 11 signatures, 6 signatures (Multigene, Bcell, Novel1, Novel2, MBC and MAGEA) were derived from TNBC, two (IR7 and Tcell) from ER-negative patients, three (SDPP, LM and IGS) from a mixed subtype patients. The cluster with higher expression had a better overall survival rate however with a modest significance (p = 0.14). (TIFF 640 kb) [file 12885_2018_4388_MOESM14_ESM.tiff]

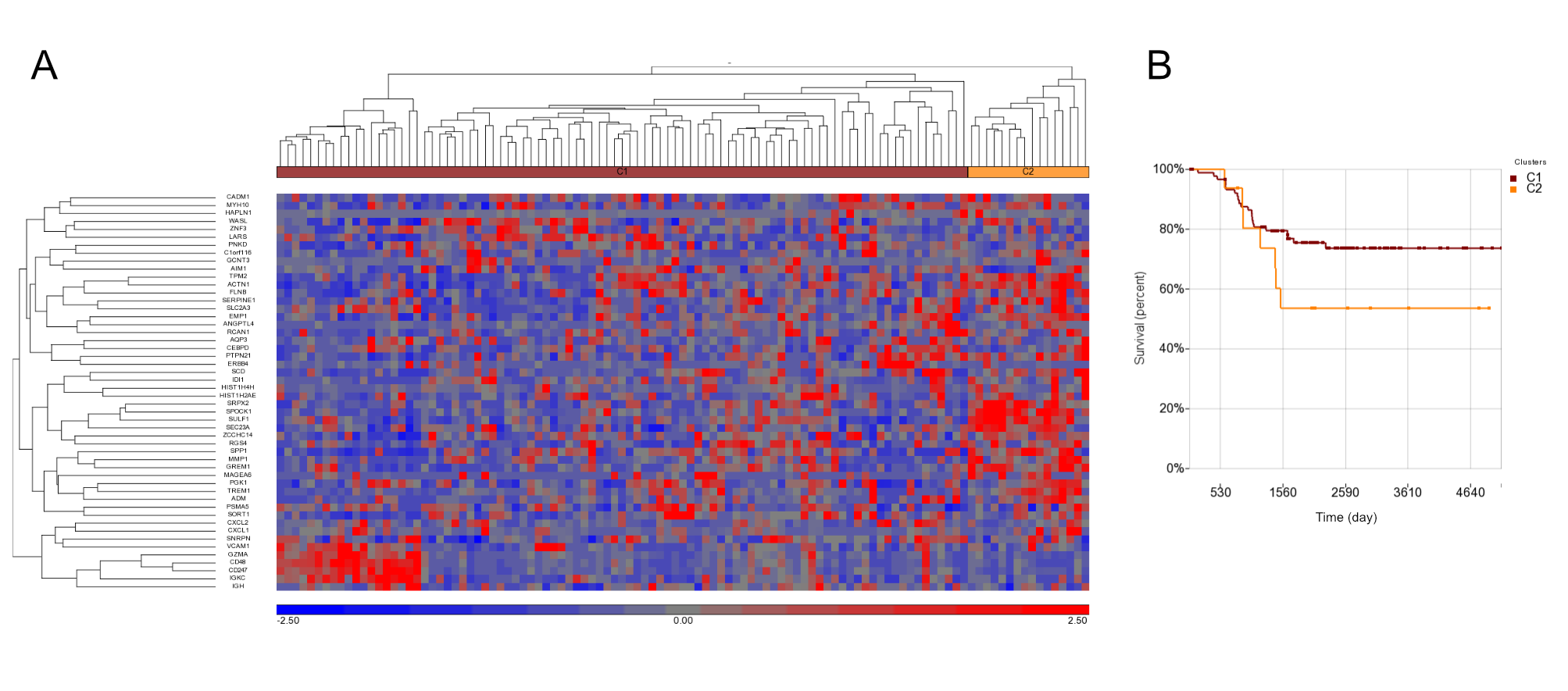

Supplement: Supplementary file 15 — Figure S7. Building TNBC subtyping classifier in GSE58812 using common genes from TNBC signature group. Six signatures (Multigene, Bcell, Novel1, Novel2, MBC and MAGEA) were derived from TNBC, two (IR7 and Tcell) from ER-negative patients, three (SDPP, LM and IGS) from a mixed subtype patients. These signature shared more common genes with each other than they shared with others. Thus genes of this 11 signatures were pooled together. Sixty-four genes overlapped in at least two of these 11 signatures were used to classify 107 TNBC patients from GSE58812 dataset into two clusters. The cluster with higher expression had a better overall survival rate however with a modest significance (p = 0.13). (TIFF 543 kb) [file 12885_2018_4388_MOESM15_ESM.tiff]
