# Supplementary material for: Genes and functions from breast cancer signatures
Source: BMC Cancer. 2018 Apr 27;18:473. doi: 10.1186/s12885-018-4388-4 (PMC5921990; doi:10.1186/s12885-018-4388-4)

Additional File 13: Fig S5

A. ER+/Node- versus normal

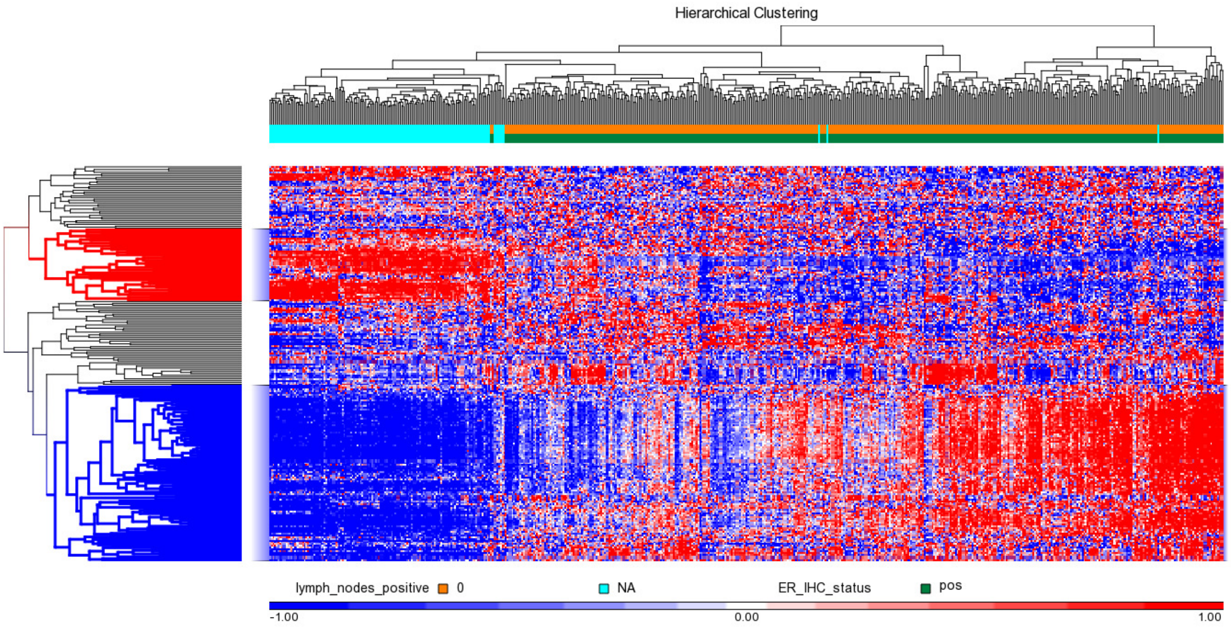

B. LumA versus normal

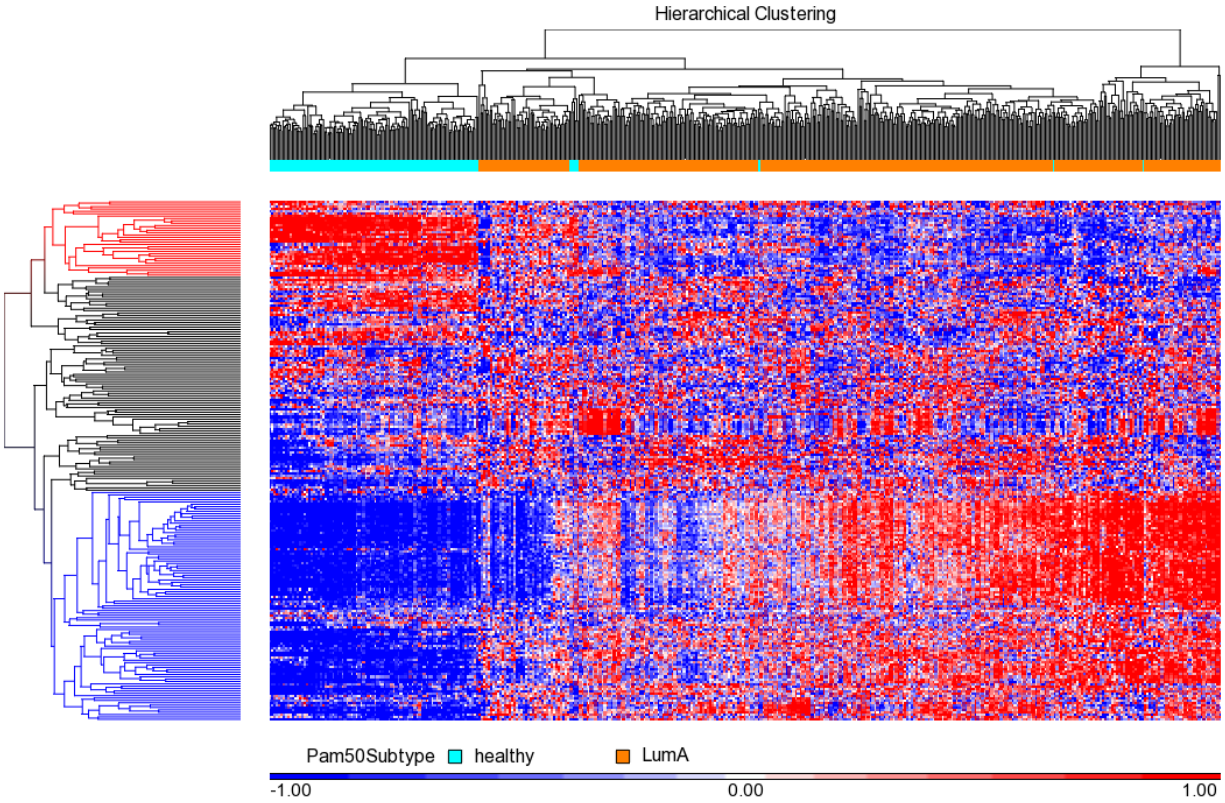

C. LumB versus normal

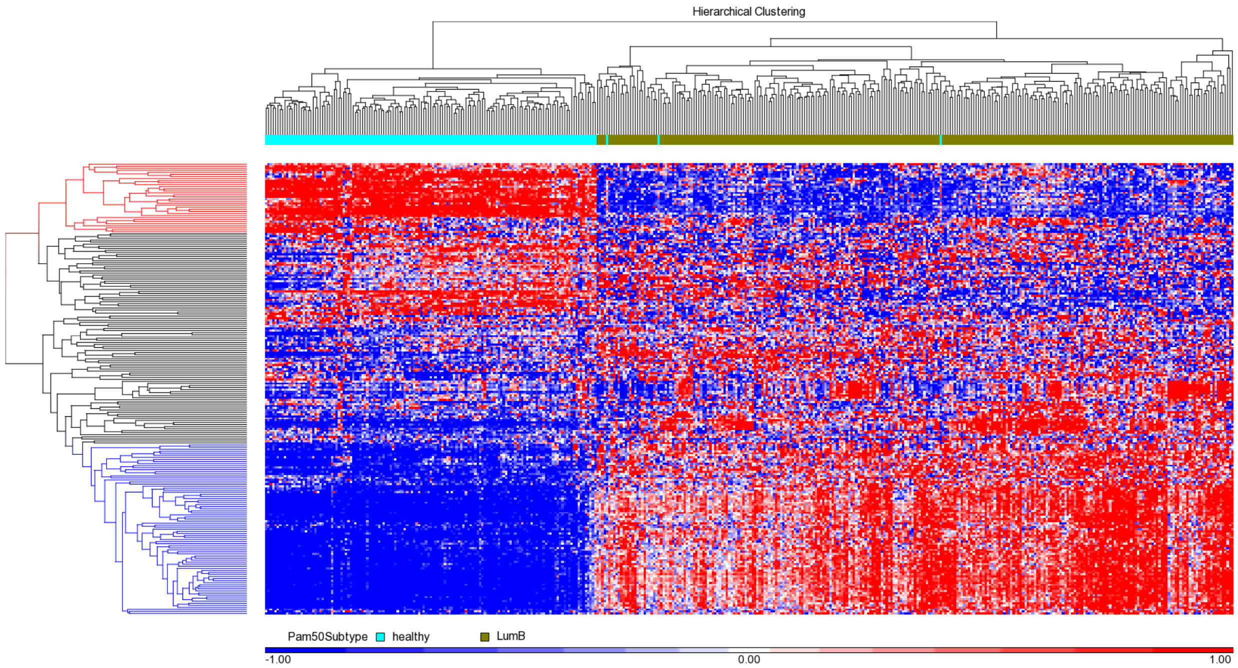

D. Her2 versus normal

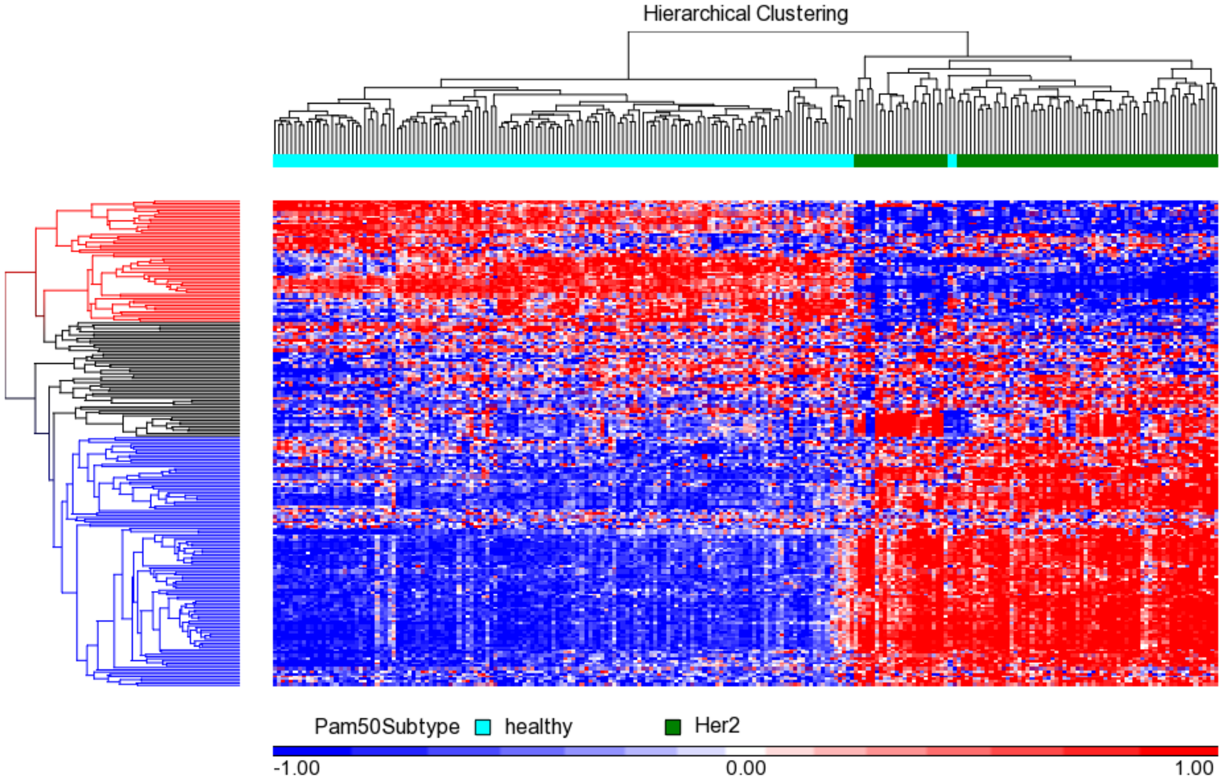

E. Basal versus normal

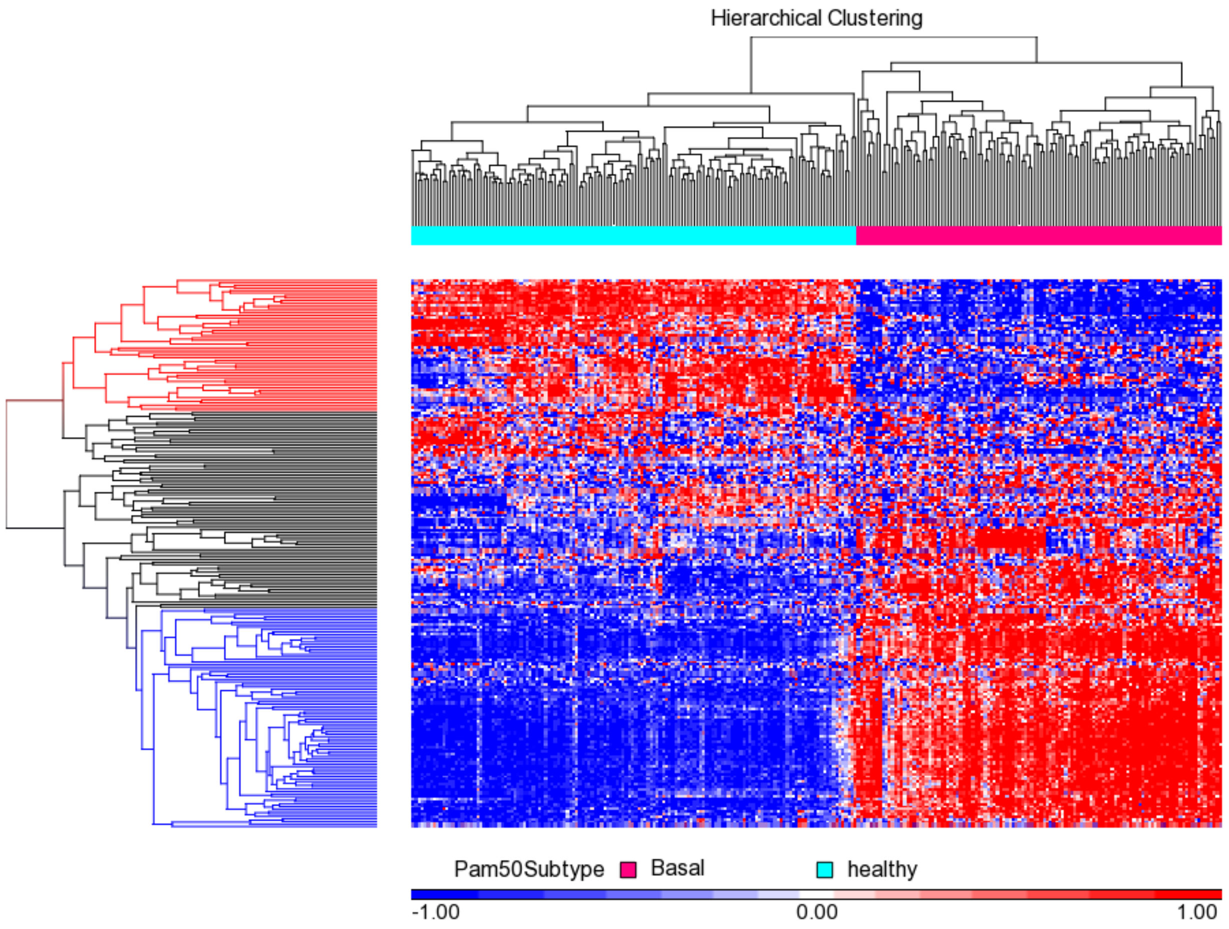

Supplement: Supplementary file 13 — Figure S5. Selection of Yin and Yang gens for different subtypes. The 220 common signature genes expression data of various cancer subtypes were extracted from METABRIC expression data set. The genes (rows) were clustering among each subtype (columns) and the normal samples (A, B, C, D, E). The contrast clusters were selected as Yin genes (in blue) and Yang (in red) genes. (PDF 1554 kb) [file 12885_2018_4388_MOESM13_ESM.pdf]
